# The host susceptibility/resistance-related genes and gut microbial characteristics in *Salmonella pullorum*-infected chickens

**DOI:** 10.1128/spectrum.00392-24

**Published:** 2025-03-03

**Authors:** Jinmei Ding, Jianshen Zhu, Hao Zhou, Kaixuan Yang, Chao Qin, Yaodong Zhang, Chengxiao Han, Lingyu Yang, Chuan He, Ke Xu, Yuming Zheng, Huaixi Luo, Kangchun Chen, Wenchuan Zhou, Shengyao Jiang, Jiajia Liu, Wenqi Zhu, Qing Niu, Zhenxiang Zhou, Shaohui Wang, Shengqing Yu, Qizhong Huang, He Meng

**Affiliations:** 1 Shanghai Key Laboratory of Veterinary Biotechnology, Department of Animal Science, School of Agriculture and Biology, Shanghai Jiao Tong University 12474 , Shanghai, China; 2 Animal Husbandry and Veterinary Research Institute, Shanghai Academy of Agricultural Science, Shanghai, China; 3 Shanghai Veterinary Research Institute, the Chinese Academy of Agricultural Sciences, Shanghai, China; University of California, Davis, San Bernardino, California, USA

**Keywords:** pullorum disease, *Salmonella pullorum*, gut microbiota, susceptibility/resistance, genome-wide association analysis

## Abstract

Pullorum disease is a bacterial disease caused by *Salmonella pullorum* in chickens, which is characterized by gastrointestinal infection and diarrhea. In traditional perspectives, research on pullorum disease primarily focused on clinical symptoms, epidemiological characteristics, and the pathogenic sites. This study, however, approaches the subject from the standpoint of host genetic basis and gut microbiota. For the positive and negative offspring chicks, which are the offspring of positive roosters and hens and negative roosters and hens, respectively, we conducted whole-genome association analysis and identified 195 SNPs and 79 significant InDels on the host genome that were associated with susceptibility/resistance to pullorum disease. A total of 77 genes were annotated, among which *MYH7, ATP2A3*, and *CACNA1S* exhibited variations in the exons. After infection with *S. pullorum*, the diversity and community structure of the gut microbiota in the chicken also underwent significant changes. *Lactobacillus, Escherichia_Shigella,* and *Klebsiella* were dominant bacteria in the dead negative offspring chicks with significantly higher abundance compared to the survival negative offspring chicks. These significant changes in host genome and bacterial abundance suggest that they may be associated with the susceptibility/resistance of pullorum disease.

**IMPORTANCE:**

Pullorum disease can be transmitted vertically and horizontally. Population purification and antibiotic treatment are the main methods for preventing and treating this disease, but they are associated with issues, such as high cost, poor accuracy, bacterial resistance, and overused antibiotics. In traditional perspectives, research on pullorum disease primarily focused on clinical symptoms, epidemiological characteristics, and the pathogenic sites. This study, however, approaches the subject from the standpoint of host genetic basis and gut microbiota. Using the genome-wide association analysis and microbiome comparison analysis, with chicken death and survival following *Salmonella pullorum* infection as phenotypes, we identified significant genetic variations (e.g., *MYH7, ATP2A3*, and *CACNA1S*) and gut microbiota (e.g., *Lactobacillus, Escherichia_Shigella, Bacillus*, and *Enterococcus_cecorum*) that may relate to susceptibility/resistance of pullorum disease. These results indicate that the infection of chickens with *S. pulloru*m and the achievement of vertical transmission may be related to the host genome and gut microbiota.

## INTRODUCTION

Poultry plays a vital role in the global agricultural economy, serving as a primary source of protein for humans. In 2024, the worldwide production of eggs and meat from poultry has reached approximately 9.2 billion and 103 million tons, respectively (1–3). However, this industry faces significant threats from various diseases, including viral, bacterial, and parasitic infections. One such bacterial disease that poses a significant risk to poultry is pullorum disease, which is caused by *Salmonella pullorum*. This disease primarily affects chickens that are less than 20 days old, resulting in high morbidity and mortality rates (4). Salmonellosis has become a major economic and public health problem in the poultry industry in most developing countries due to its widespread epidemic, difficulty in complete elimination, and food safety concerns (5–8).

*S. pullorum* can spread both vertically and horizontally (9). Once it enters the chicken’s intestines, it starts to multiply, breaks through the intestinal mucosa, and utilizes dendritic cells to transport the bacteria to the mesenteric lymph nodes. From there, it invades the blood, liver, spleen, and other organs through the lymphatic system. The bacteria can establish long-term colonization in the liver and spleen of chickens, turning them into negative carriers that transmit the infection horizontally to other chickens (10). Additionally, *S. pullorum* can enter a rooster’s semen or a hen’s reproductive tract, enabling vertical transmission of the disease (11). The presence of both vertical and horizontal modes of transmission contributes to the continuous spread of *S. pullorum*. Currently, two main methods are employed in poultry production to prevent pullorum disease. The first method involves purifying breeding chickens based on the results of an antibody detection test, called the whole blood plate agglutination test. The second method relies on the use of antibiotics to control the spread and morbidity of pullorum disease. While these methods are effective in preventing the disease in chickens, they come with their own set of problems, such as high purification costs, limited accuracy, bacterial drug resistance, and the overuse of antibiotics (12). Traditionally, research on pullorum disease has primarily focused on clinical symptoms, epidemic characteristics, and the pathogenic genes of (13) *S. pullorum*. Little attention has been given to understanding how the host influences the *S. pullorum* infection. In reality, the occurrence of *S. pullorum* infection can vary significantly among different breeds of chickens and even among individuals within the same breed (14). Even in a shared environment, some chickens may become infected, while others remain unaffected. This variability could be attributed to genetic factors among individuals. Therefore, investigating the genetic basis of the host will be valuable in uncovering the molecular mechanisms of *S. pullorum* infection within the chicken population.

Elmer Roberts demonstrated, for the first time, that chickens were resistant/susceptible to *S. pullorum* after inoculating 29,000 chickens with *S. pullorum* in 1935 (15). Single-comb White Leghorns have a higher natural resistance to *S. pullorum* than Rhode Island Reds, regardless of whether they have been infected with *S. pullorum* (16). Thus, different chicken lines or breeds have different resistance/susceptibilities to pullorum disease (17). In the study of *S. pullorum* resistance/susceptibility genes, the expressions of *TLR4*, *MyD88*, *TRAF6*, and *NF-κB* in spleen and *TLR2*, *TLR21,* and *AvBD6* in intestinal tissue were significantly up-regulated in 3 day-old chickens of oral-administration *S. pullorum* (18–20). *NLRC5* negatively regulates the NF-κB pathway in *S. pullorum* carrier (21). Chicken infection with *S. pullorum* can also activate NOD1 receptor signaling, affecting the expressions of *RIPK2*, *NF-κB*, *MAPK11*, *IL-1b*, and *IL-8* (22). A comparison of the *MyD88* coding region in infected and uninfected chicks showed that SNPs in this region were significantly associated with *S. pullorum* infection (23). These studies have focused on gene expression through RT-PCR or transcriptome, and the results may be influenced by individual growth and development and *S. pullorum* infection. It is urgent to consider the problem from the perspective of the host genome in the study of susceptibility/resistance to *S. pullorum*. In 2019, we identified 43 significant SNPs associated with chicken *S. pullorum* infection through double-digest genotyping-by-sequencing (24). Through 600 k high-density chip sequencing analysis, Li et al. found that *FBXW7* and *LRBA* may be the resistance genes of *S. pullorum,* and *TRAF3* and *gga-mir-489* may be candidate genes potentially associated with whether an individual carries *S. pullorum* (25). But, our understanding of the host susceptibility/resistance-related genes and gut microbial characteristics following *S. pullorum* infection remains limited.

This study employed genome resequencing and gut microbial 16S rRNA sequencing to investigate the genetic basis and gut microbial characteristics of *S. pullorum*-infected chicks. Our objective was to identify genetic variations and gut microbes that play a significant role in determining susceptibility or resistance to *S. pullorum*. These findings are expected to provide a basic reference for the study of molecular mechanisms, the purification, the breeding for disease resistance, and the intervention and adjustment of gut microbiota in *S. pullorum*-infected chickens.

## RESULTS

### Characteristics of *S. pullorum* infection in chicks

Based on the whole blood plate agglutination test, we examined the prevalence of *S. pullorum* in Xin Pudong chickens. Testing was performed monthly for three consecutive months. A total of 23 chickens tested positive three times in a row, including six roosters and 17 hens. Ninety-eight chickens tested negative three times in a row, including 12 roosters and 86 hens. For these individuals, the fresh blood or cultured fecal anal swab solutions were coated on SS AGAR medium for overnight culture to detect the presence of *S. pullorum*. No *S. pullorum* was detected in these individuals. A total of 611 negative offspring chicks were obtained by breeding negative roosters and negative hens, while 227 positive offspring chicks were obtained by breeding positive roosters and positive hens.

A total of 120 chicks were selected for the determination of LD50, including 60 negative offspring chicks and 60 positive offspring chicks. Six different concentrations of SP1218 suspensions (5 × 10^9^, 5 × 10^8^, 5 × 10^7^, 5 × 10^6^, 5 × 10^5^, and 5 × 10^4^ CFU/mL) were inoculated on 3 day-old chicks of 10 negative or positive offspring, respectively. According to the formula of LD50, the appropriate LD50 for the challenge test is 6.3 × 10^6^ CFU (Table S1).

**Fig 1:**
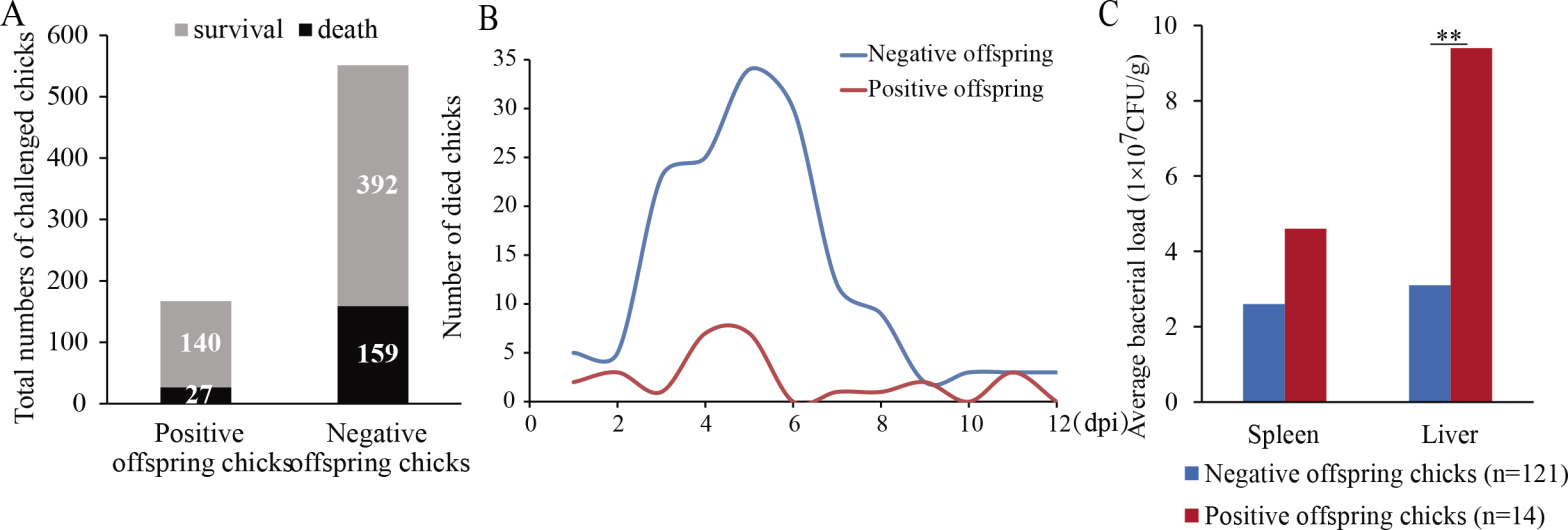
(**A**) Mortality of positive and negative offspring chicks, (**B**) the number of dead chicks infected with *S. pullorum*, and (**C**) liver and spleen bacterial load of positive and negative offspring chicks, **P < 0.01.

In the challenge test, 718 3 day-old chicks (including 167 positive offspring chicks and 551 negative offspring chicks) were injected with SP1218 into their leg muscle, that is, 0.2 mL/chick. After being challenged, some chicks showed the phenomenon of anal paste, even dying by anal paste (Fig. S1). At the same time, the autopsy of the deceased individuals showed significant liver and spleen lesions (Fig. S2). Up to 13 days post-infection (dpi), the mortality rate of positive offspring chicks was approximately 16%, with a total of 27 dead chicks. The negative offspring chicks had a mortality rate of about 29%, with a total of 159 dead chicks (Fig. 1A). The peak of deaths occurred on 3 to 7 dpi after *S. pullorum* infection (Fig. 1B).

Liver samples from 100 dead and three alive negative offspring chicks were randomly selected to detect the presence of SP1218. SP1218 was detected in 89 dead samples and one surviving sample by PCR (Fig. S3). Three samples with PCR strips were randomly selected for gluing recovery and sequenced. The results showed that the strain detected in the liver was SP1218 (Fig. S4; Table S4).

Liver and spleen samples were collected to calculate the bacterial load, including 121 dead negative offspring chicks, 14 dead positive offspring chicks, and 48 alive negative offspring chicks. The bacterial load in the liver and spleen of dead positive offspring chicks was 9.4 × 10^7^ and 4.6 × 10^7^ CFU/g, respectively, while that in the liver and spleen of dead negative offspring chicks was 3.1 × 10^7^ and 2.6 × 10^7^ CFU/g, respectively (Fig. 1C). Among them, the liver bacterial load of dead negative offspring chicks was lower than that of dead positive offspring chicks, and the difference was very significant (*P* < 0.01). The bacterial load in the spleen of dead negative offspring chicks was also lower than that of dead positive offspring chicks, but the difference was not significant. Meanwhile, the bacterial load in the liver and spleen of 48 alive individuals was too low to count.

### Significant genetic variation associated with susceptibility/resistance traits

Seventy-eight dead (SP1218 was detected in their livers) and 71 surviving negative offspring chicks were selected to collect liver samples for genome resequencing. Based on the sequence localization results in the reference genome, 31,047,097 SNPs and 5,134,678 InDels were detected in 149 samples. A total of 8,047,152 SNPs and 977,909 InDels were obtained by population filtering. We performed a genome-wide association study using GEMMA software. In the analysis, death and survival were used as phenotypes. GEMMA could identify the genotypes and phenotypes of the PLINK binary ped file format. First, we used PLINK to further filter 8,047,152 SNPs. The criteria are as follows: the deletion rate of a single sample should not be greater than 50%; the deletion rate of a single locus should not be greater than 20%; and the minimum allele frequency should be less than 0.05. That is, individuals with a positive rate of less than 50% were eliminated; SNPs with a positive rate of more than 80% were retained; and the loci with minimum allele frequency less than 0.05 were deleted.

**Fig 2:**
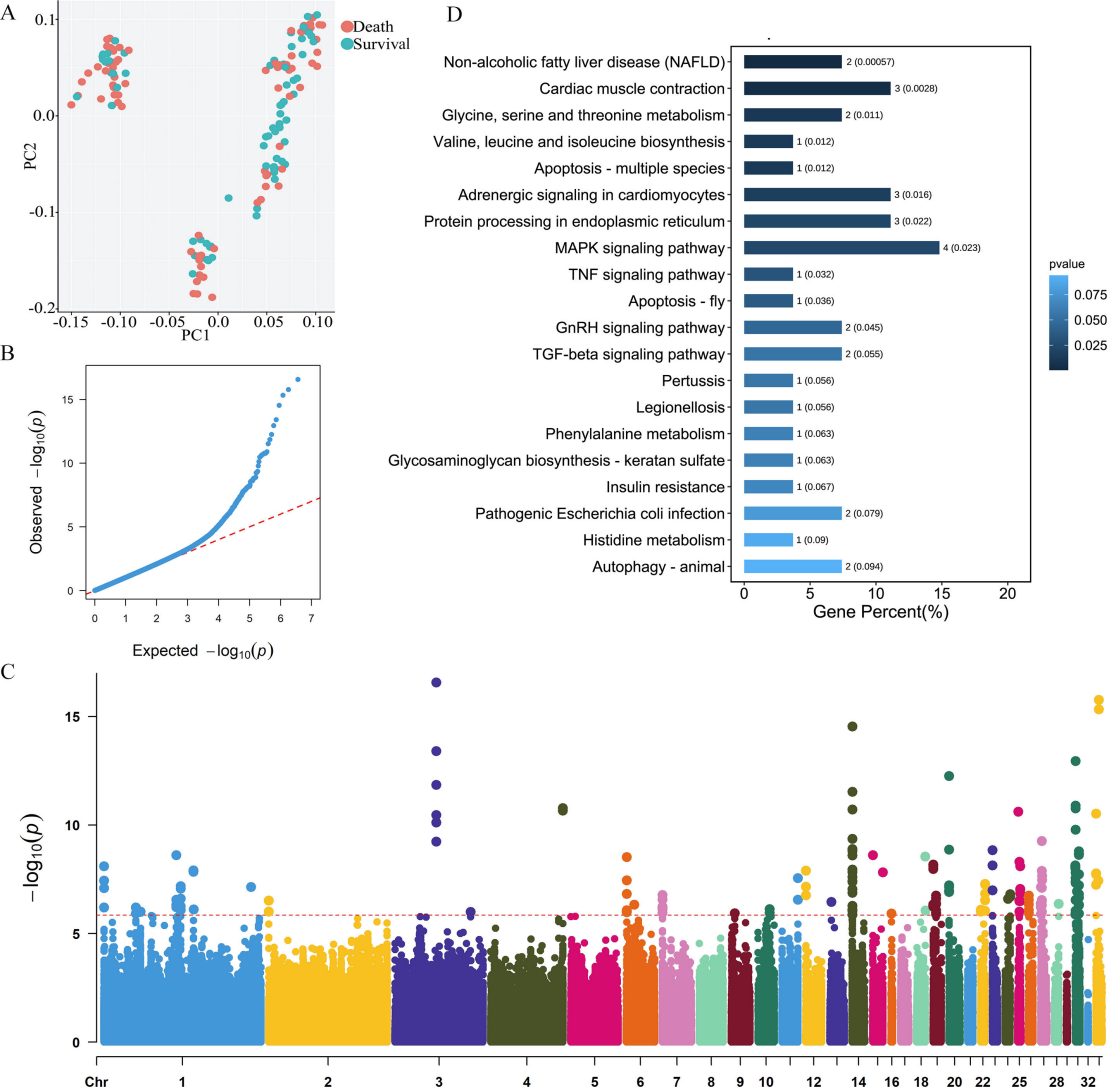
(**A**) Sample population stratification, (**B**) quantile–quantile plot of susceptibility or resistance of *S. pullorum*, (**C**) Manhattan plot of SNP distribution on chicken chromosome: the horizontal axis is the chromosome; the vertical axis is the *P* value of Log10; and the significant association threshold of 1.38 × 10^−6^ is represented by the red dotted line; and (**D**) Kyoto Encyclopedia of Genes and Genomes (KEGG) pathway enrichment: the horizontal axis represents the gene number and the ratio of the candidate genes vs all genes involved in that specific pathway, while the vertical axis represents the KEGG pathway.

The principal component of the population genetic background calculated based on SNPs showed that there was no significant bias between the genetic backgrounds of the dead and surviving groups (Fig. 2A). Then, the correlation matrix was calculated based on phenotype and genotype, and genome-wide association study was performed, resulting in 3,616,707 SNPs. The quantile–quantile (Fig. 2B) and Manhattan plots (Fig. 2C) are drawn with this result. The loci in the lower left corner of the quantile–quantile plot (Fig. 2B) indicate that our model is reasonable, and those with a higher significance in the upper right corner may be candidate loci related to traits. The threshold of the Manhattan plot (Fig. 2C) is *P* = 1.38 × 10^−6^. We obtained 195 significant SNPs and 79 significant InDels (Table 1; Tables S3 and S4). There were multiple significant SNPs clustered into clusters, and the clusters of seven or more SNPs on a single chromosome accounted for 87% of the total number of significant SNPs. For example, there were 21, 21, 23, 11, 16, and 27 significant SNPs clustered on chromosomes 1, 14, 19, 25, 27, and 31, respectively. The SNP most significantly associated with *S. pullorum* infection in chicks was located at 51,806,310 bp on chromosome 3 (Fig. 2C; Table S3).

**TABLE 1:** Significantly different SNPs^*a*^

Chr	Position	Ref/alt	*P* value	Chr	Position	Ref/alt	*P* value	Chr	Position	Ref/alt	*P* value
3	51806310	A/G	2.69E−17	14	311098	A/T	2.50E−09	27	1788139	G/A	2.24E−08
33	3531645	T/G	1.71E−16	18	11033965	T/A	2.81E−09	14	350317	A/G	2.49E−08
33	3531614	G/A	4.72E−16	6	1181342	C/A	3.02E−09	31	206565	G/A	2.79E−08
14	412285	C/T	2.92E−15	25	2139225	A/G	5.01E−09	11	20016948	C/G	2.86E−08
3	51806342	A/G	3.97E−14	19	292549	T/C	6.63E−09	27	1788135	T/A	3.44E−08
31	207291	T/G	1.14E−13	19	292552	G/A	6.63E−09	27	1788138	A/C	3.44E−08
20	34774	G/T	5.64E−13	19	292564	G/A	6.63E−09	6	1154572	G/C	3.52E−08
3	51806265	C/T	1.43E−12	19	292567	T/C	6.63E−09	33	2994742	C/T	3.66E−08
14	340154	G/A	2.98E−12	31	190424	G/C	7.22E−09	1	135889	C/T	3.71E−08
31	205379	G/A	1.27E−11	23	11861	G/A	7.31E−09	14	228321	A/C	3.94E−08
4	90708600	G/A	1.68E−11	1	125067	A/G	7.89E−09	31	199927	G/A	4.05E−08
31	215142	T/G	1.71E−11	25	3085069	A/G	8.04E−09	14	340301	A/T	4.13E−08
14	399056	T/G	1.94E−11	19	292588	A/G	8.24E-09	31	201506	T/G	4.30E−08
4	90708634	C/T	2.19E−11	31	50647	C/A	8.56E−09	31	191728	G/C	4.65E−08
25	563863	G/A	2.46E−11	31	190950	G/T	9.84E−09	22	5399796	A/T	5.28E−08
33	11964	G/A	2.98E−11	19	292561	A/G	1.08E−08	22	5376078	A/G	5.49E−08
3	51806274	A/C	3.47E−11	19	292562	C/T	1.08E−08	33	12036	C/A	5.70E−08
3	51806368	C/T	7.81E−11	14	311094	T/G	1.13E−08	20	24866	C/T	6.05E−08
31	215143	C/T	1.67E−10	1	1.13E + 08	C/G	1.23E−08	31	4540692	T/C	6.07E−08
14	349498	G/A	4.42E−10	12	250345	A/T	1.29E−08	1	96568933	G/A	6.59E−08
27	1708279	T/C	5.46E−10	27	1710211	C/A	1.31E−08	12	209600	G/T	7.03E−08
3	51806396	C/A	5.92E−10	1	1.13E + 08	A/G	1.45E−08	1	1.85E + 08	T/C	7.27E−08
14	412254	C/A	1.29E−09	14	350319	T/G	1.47E−08	27	517515	C/G	7.29E−08
20	23948	C/T	1.37E−09	14	397675	G/C	1.47E−08	20	19014	T/C	7.33E−08
23	7619	C/T	1.46E−09	15	13017597	C/A	1.53E−08	27	1571595	T/C	7.90E−08
14	311372	G/C	1.56E−09	31	203405	T/C	1.56E−08	1	135904	A/G	8.09E−08
31	4534334	C/A	1.65E−09	33	12006	A/G	1.70E−08	1	96568937	A/T	8.50E−08
31	4534084	T/C	2.05E−09	31	4662960	A/G	1.84E−08	25	3083201	A/G	8.91E−08
31	4534296	T/G	2.40E−09	33	12113	G/A	1.86E−08	14	350313	A/T	9.16E−08
1	90840932	G/A	2.45E−09	27	1788137	G/A	2.24E−08	25	3088135	G/C	9.61E−08
15	278779	G/T	2.47E−09								

^*a*^ Only SNPs with *P* < 1 × 10^−7^ are shown in the table.

### Genes related to susceptibility/resistance traits and their functions

Sixty genes were identified by gene annotation of 195 significant SNPs (Table 2; Table S3). The most significant SNP related to susceptibility/resistance traits of *S. pullorum* is about 155 kb away from the gene *GTF2H5* (general transcription factor IIH subunit 5). An SNP annotation on chromosome 1 is on the intron of the *CNTN5* (contactin 5). On chromosome 19, one SNP is annotated on the exon of *MYH7* (myosin, heavy chain 7, cardiac muscle, beta). Twelve SNPs are annotated on the exon of *ATP2A3* (ATPase, Ca2+ unexpectedly, ubiquitous). Among them, two SNPs of 3,440,011 and 3,449,102 bp are nonsynonymous mutations, from G to C in exon eight and from T to G in exon 15. On chromosome 26, seven SNPs are located on the exon of the *CACNA1S* (calcium voltage-gated channel subunit alpha1 S). The SNPs on 276,599, 276,604, and 276,614 bp are nonsynonymous mutations, which were mutated from G to T, G to C, and A to G, respectively, on exon 25 (Table 2; Table S3). Seventy-nine significant InDels were annotated to 41 genes (Table S4). The most significant locus was on the 11,154,013 bp of chromosome 18, where a 35-base deletion occurred. The nearest gene was *GRB2* (growth factor receptor bound protein 2). Two InDels on chromosome 3, one at 11,882,630 bp, are annotated on the intron of the gene *PPP3R1* (protein phosphatase three regulatory subunit B, alpha). For one at 31,492,722 bp, the annotation is on the intron of the *TTC27* (tetratricopeptide repeat domain 27) gene.

**TABLE 2:** SNPs annotated to gene exons significantly associated with *S. pullorum* infection

Chr	Mutating location (bp)	Ref/alt	*P* value	Genes
19	58530	T/G	4.58E−07	MYH7
19	3439993	T/C	1.19E−06	ATP2A3
19	3440003	A/C	1.19E−06	ATP2A3
19	3440011	G/C	1.07E−06	ATP2A3
19	3440026	A/G	1.80E−07	ATP2A3
19	3440027	C/T	1.80E−07	ATP2A3
19	3440032	G/C	2.53E−07	ATP2A3
19	3440041	T/C	4.45E−07	ATP2A3
19	3440053	T/C	2.35E−07	ATP2A3
19	3440056	C/T	4.04E−07	ATP2A3
19	3440065	C/G	2.50E−07	ATP2A3
19	3445965	G/A	1.28E−06	ATP2A3
19	3449102	T/G	8.40E−07	ATP2A3
26	275357	T/C	8.33E−07	CATSPER2
26	276574	T/C	8.83E−07	CATSPER2
26	276599	G/T	2.86E−07	CATSPER2
26	276601	G/C	2.86E−07	CATSPER2
26	276604	G/C	1.79E−07	CATSPER2
26	276614	A/G	4.52E−07	CATSPER2
26	276631	T/C	1.15E−06	CATSPER2

The enrichment analysis of these genes showed that 27 genes were enriched in 55 Kyoto Encyclopedia of Genes and Genomes (KEGG) metabolic pathways, of which 11 pathways were significantly different (Fig. 2D). The most significant is that *CASP14* and *MLXIPL* participate in the metabolism of nonalcoholic fatty liver disease (*P* = 0.0006). Then, there are cardiac muscle contractions (*P* = 0.0028) and adrenergic signaling in cardiomyocytes (*P* = 0.016), which are participated in by *CACNA1S*, *ATP2A3*, and *MYH7*. Four genes, namely, *GRB2*, *CACNA1S*, *RPS6KA*, and *DUSP7*, are involved in the MAPK signaling pathway (*P* = 0.0229). *CASP14* is involved in apoptosis and TNF signaling pathway (*P* = 0.0322). In addition, *CASP14* is involved in pertussis (*P* = 0.0557), legionellosis (*P* = 0.0557), Alzheimer’s disease (*P* = 0.101), and pathways in cancer (*P* = 0.2499). *CASP14* and *ABCF2* are involved in the pathogenic *Escherichia coli* infection (*P* = 0.0792). *CASP14* and *S100A10* are involved in *Salmonella* infection (*P* = 0.2076). Although these pathways are not significantly different, they suggest that *CASP14* may be an important candidate gene for disease (Table S5).

### *S. pullorum* infection changes the diversity of bacteria

According to the dead and surviving chicks obtained after the challenge experiment, 20 of 78 dead negative offspring chicks (ND), 20 of 71 survival negative offspring chicks (NS), five dead positive offspring chicks (PD), and five survival positive offspring chicks (PS) were selected from the resequencing samples. The cecal contents of the above 50 chicks were collected for 16s rRNA sequencing to analyze the gut microbial characteristics after *S. pullorum* infection.

**Fig 3:**
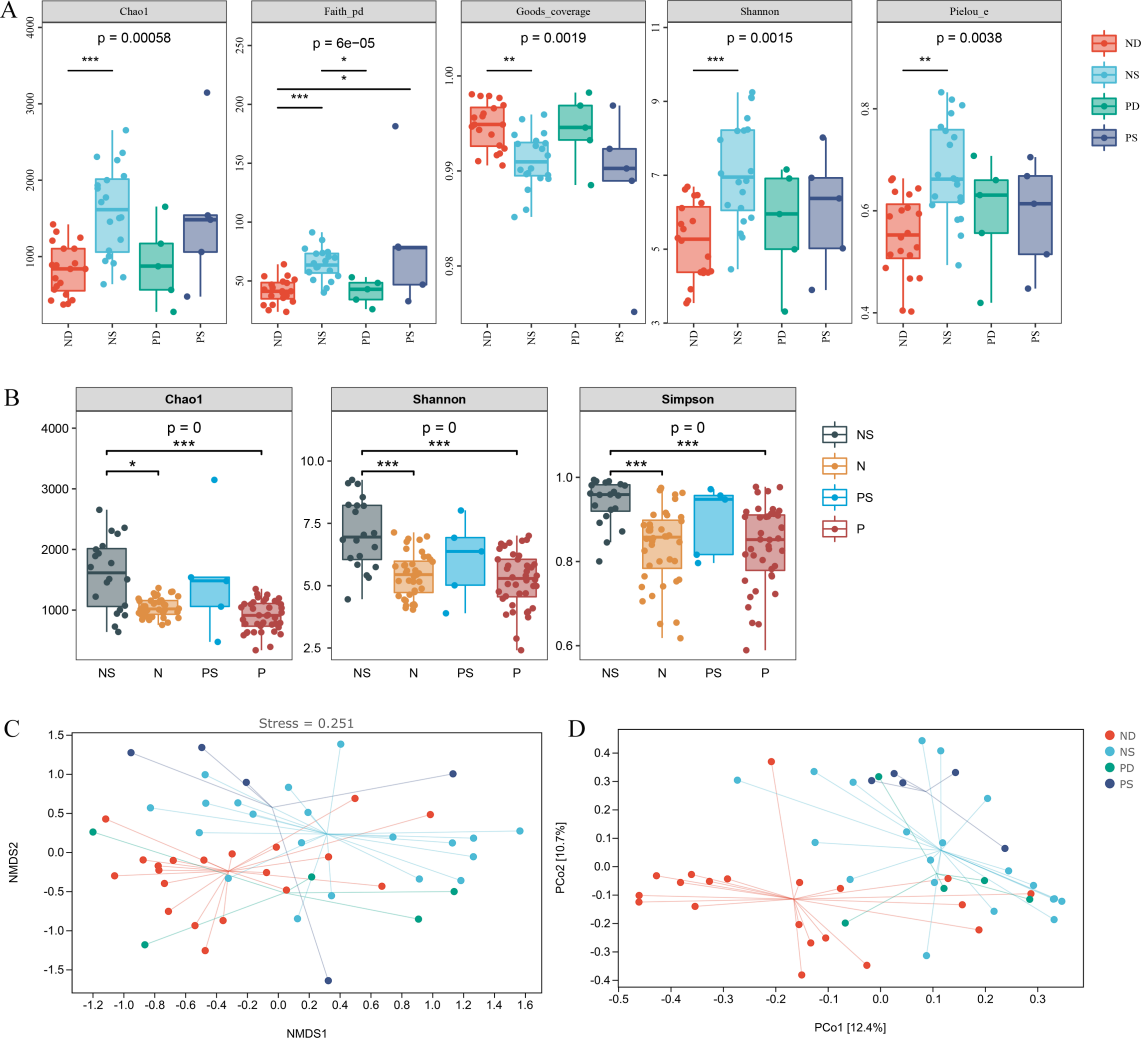
(**A**) Alpha diversity index for each group. (**B**) Comparison of the gut microbial alpha diversities of chicks before and after the *Salmonella pullorum* challenge. The horizontal axis of A and B is the group label, while the vertical axis of A and B is the value of the corresponding alpha diversity index. The asterisk is the significance marker. (**C**) Nonmetric multidimensional scaling (NMDS); the distance between points indicates the difference in microbial communities between samples. (**D**) In the principal coordinates analysis (PCoA), the percentage is the proportion of sample difference data (distance matrix).

To evaluate the influence of *S. pullorum* infection on gut microbial diversity, we compared the alpha diversity indices of Chao1, Shannon, Faith’s PD, Pielou’s evenness, and Good’s coverage. The results showed that the microbial richness, diversity, and evenness were higher in NS than in ND, while the coverage was opposite (Fig. 3A). There was no significant difference between the other groups. In summary, *S. pullorum* infection in dead chicks significantly reduced the abundance and diversity of gut microbiota.

We also compared the gut microbial diversities of chicks before and after *S. pullorum* infection (Fig. 3B). Chick samples before *S. pullorum* infection are from our previous study (26), including 40 negative offspring chicks obtained by breeding negative roosters and hens (N) and 40 positive offspring chicks obtained by breeding positive roosters and hens (P). The comparison showed that the gut microbial diversity and richness in NS were significantly higher than that in groups N and P. There was no significant difference between P and PS (Fig. 3B). This result indicates that the gut microbial abundance and diversity of chicks were significantly increased in order to fight off *Salmonella pullorum* infection.

We studied the heterogeneity of the species composition between samples through beta diversity. The nonmetric multidimensional scaling (NMDS) and principal coordinates analysis (PCoA) showed that there was a large difference in microbial community diversity between the surviving and deceased groups, especially between the NS and the ND (Fig. 3C and D). This is consistent with the previous analysis of alpha diversity.

### Gut microbiota associated with *S. pullorum* infection in chickens

**Fig 4:**
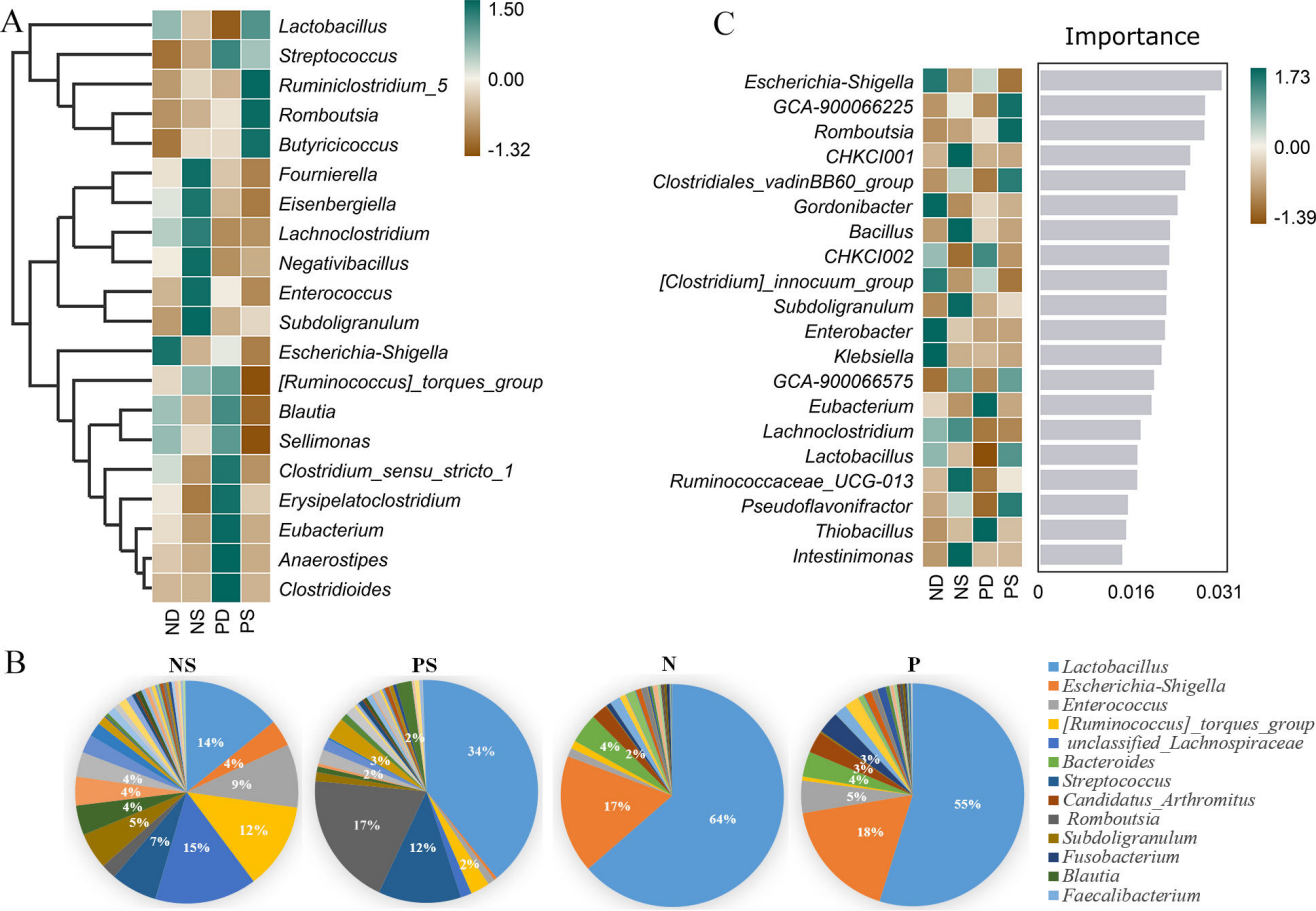
(**A**) Heatmap of the microbial composition between groups at the genus level; the color ranges from dark brown to dark green in the heatmap and indicates the abundance of bacteria ranging from low to high. (**B**) Comparison of the gut microbial compositions in chicks before and after the bacterial challenge at the genus level. (**C**) Random forest analysis. The horizontal coordinate of the bar graph scores the importance of the species to the classifier model, and the vertical coordinate is the taxon name at the genus level. The heat map shows the distribution of the bacterial abundance in each group. The importance of species to the model decreases from top to bottom. These most important species can be regarded as markers of differences between groups.

Analysis of the gut microbial composition in chicks infected with *S. pullorum* showed that Firmicutes was the most dominant phylum in each group, accounting for 79.08% in ND, 95.47% in NS, 90.51% in PD, and 90.15% in PS. The second phylum was Proteobacteria, which accounted for 20.24% in ND, 3.95% in NS, 9.01% in PD, and 5.99% in PS (Fig. S5). At the genus level, *Escherichia_Shigella*, *Lactobacillus*, *Lachnoclostridium*, *Blautia*, *Sellimonas*, and *Clostridium_sensu_stricto_1* were the most abundant genera in ND (Fig. 4A). *Fournierella*, *Eisenbergiella*, *Lachnoclostridium*, *Negativibacillus*, *Enterococcus*, *Subdoligranulum*, and *[Ruminococcus]_torques_group* were the most abundant genera in NS. The abundance of *Streptococcus*, *Ruminococcus_torques_group*, *Blautia*, *Sellimonas*, *Clostridium_sensu_stricto_1*, *Erysipelatoclostridium*, *Eubacterium*, *Anaerostipes*, and *Clostridioides* were enriched in PD. Meanwhile, *Lactobacillus*, *Streptococcus*, *Ruminiclostridium_5*, *Romboutsia*, and *Butyricicoccus* had a higher abundance in PS.

Analysis of different gut microbiota among multiple groups revealed that 51 bacteria showed significant differences among the four groups (Table S6). At the phylum level, Firmicutes was significantly higher in NS than the others (*P* < 0.05), and Proteobacteria in ND was significantly higher than that of the others (*P* < 0.05). Compared with the other three groups, the genera of *Escherichia_Shigella* and *Klebsiella* in ND were significantly increased (*P* < 0.05). The abundance of *Enterococcus_cecorum* and *Lactobacillus_rennini* in NS was significantly higher than that in the other groups (*P* < 0.05). The abundance of *Thiobacillus* and *Hydrogenophilaceae* in PD were significantly raised (*P* < 0.01). The abundance of *Lactobacillus_vaginalis*, *Erysipelatoclostridium_bacterium_ic1391*, and *Pseudomonas* in PS were also significantly higher than that in other groups (*P* < 0.05). Comparative analysis showed that there were 68 significantly different bacteria between ND and NS (*P* < 0.05) (Table S7). *Lactobacillus* and *Escherichia_Shigella* were significantly more abundant in ND than in NS, while *Eisenbergiella* and *Bacillus* were on the contrary (*P* < 0.05). *Lactobacillus* also was significantly higher in ND than PD, and *Eubacterium* and *Eggerthellaceae_CHKCI002* were opposite (Table S8). The abundance of *Escherichia_Shigella*, *Eubacterium*, and *Cetobacterium* in PD was significantly higher than that in PS (*P* < 0.05) (Table S9). *Eisenbergiella* and *Bacillus* were significantly higher in NS than PS (*P* < 0.05) (Table S10). They may be important genera associated with *S. pullorum* infection.

We also compared the gut microbial compositions of chicks before (groups P and N) and after (groups PS and NS) *S. pullorum* infection (Fig. 4B; Tables S10 and S11). At the genus level, groups P and N had high abundance of *Lactobacillus*. The abundance of *Lactobacillus* and *Escherichia_Shigella* in N were significantly higher than that in the NS. However, the abundance of *Enterococcus*, *Bacillus*, *Blautia*, and *Streptococcus* were significantly higher in NS than in N. It was also found that the abundance of *Escherichia_Shigella* and *Bacteroides* in *P* were significantly higher than that in PS. On the contrary, the abundance of *Erysipelatoclostridium* and *Fournierella* were significantly higher in PS. These results indicated that *S. pullorum* infection significantly changed the gut microbial composition in chicks.

In order to know which species are more significant, we analyzed species importance through the random forest analysis (Fig. 4C), which can effectively, robustly, and accurately classify microbial community, that is, effectively find marker species. The results showed that *Escherichia_Shigella*, *Gordonibacter*, *Clostridium_innocuum_group*, *Enterobacter*, *Klebsiella*, *Lachnoclostridium*, and *Lactobacillus* were important marker bacteria of ND; *Eggerthellaceae_CHKCI001*, *Bacillus*, *Subdoligranulum*, *Lachnoclostridium*, *Ruminococcaceae_UCG-013*, and *Intestinimonas* were the important markers of NS. *Eggerthellaceae_CHKCI002*, *Eubacterium,* and *Thiobacillus* were important markers of PD. *Ruminococcaceae_GCA-900066225*, *Romboutsia*, *Clostridiales_vadinBB60_group,* and *Pseudoflavonifractor* were important markers of the bacterial genera of PS (Fig. 4C).

## DISCUSSION

### The *S. pullorum* infection is related to the host genetic basis

After the chicks were challenged with *S. pullorum* in our study, the mortality rate of positive offspring chicks (16%) was lower than that of negative offspring chicks (29%), and the liver bacterial load of dead positive offspring chicks was significantly higher than that of dead negative offspring chicks. This may be because the positive offspring chicks have vertically transmitted maternal antibodies to *S. pullorum,* so when they are inoculated with *S. pullorum* again, the body has a secondary immune response, which can timely clear bacterial infection, resulting in a lower mortality. However, negative offspring chicks failed to obtain maternal antibodies, and when inoculated with *S. pullorum*, there was a strong immune response, causing a large number of chicks to die, so we observed a high mortality. At the same time, the chicks die before *Salmonella pullorum* can invade their organs, so we detected a low bacterial load in the dead negative offspring chicks. In addition, the liver and spleen bacterial loads in the survival negative offspring chicks were too low to count compared to the death group. This indicates that the surviving individuals may have been cleared of *S. pullorum* and also implies that there may be differences in the genetic background of the deceased and surviving chicks. These results suggest that whether chickens are infected with *S. pullorum* or have vertical transmission may be related to the host genome. This reminds us that in addition to considering whether chickens are or have been infected with *S. pullorum*, the host genetic basis should also be considered for *S. pullorum* susceptibility or resistance. However, we still do not know the genetic differences of the positive and negative populations and their offspring. Therefore, it is very necessary to understand the genetic mechanism of *S. pullorum*-infected chickens, which will be a new way to prevent and treat chicken pullorum disease. It is based on this idea that the whole genome association study was adopted in our research, with death and survival as the phenotype, to explore the mechanism of chicken *S. pullorum* infection and to break through the bottleneck of chicken pullorum disease purification and prevention from the perspective of genetic basis.

In this study, genomic DNA was collected for whole-genome resequencing and GWAS analysis of 3 day-old chickens infected with *S. pullorum*. By comparing the genetic variations between the dead and surviving groups, it was found that the three genes *MYH7*, *ATP2A3*, and *CACNA1S* had exon variation, among which *ATP2A3* and *CACNA1S* were nonsynonymous mutations. *MYH7* is a myosin heavy-chain expression gene that continues to be expressed in the posterior tubule of chicken development. *MYH7* mutation can lead to severe hypertrophic and dilated cardiomyopathy, which can eventually lead to heart failure (27, 28). *ATP2A3* is a magnesium-dependent ATP hydrolase that transports calcium from the cytoplasm to the sarcoplasmic/endoplasmic reticulum, participates in muscle excitation/contraction and myocardial cell contraction, maintains intracellular calcium ion homeostasis, and contributes to calcium absorption (29, 30). *ATP2A3* has been reported to have associations with severe corona virus disease 2019 heart failure, breast cancer, Parkinson’s disease, bipolar disorder, white spot disease, and muscular atrophic disease (31–33). *CACNA1S* is an important voltage-gated protein in skeletal muscle calcium ion channels, which can activate the opening of endoplasmic reticulum calcium ion release channels and increase cytoplasmic calcium ion concentration (34). In the mycobacterium tuberculosis infection, calcium homeostasis is significantly related to the expression of *CACNA1S*, and the changes in *CACNA1S* expression level increase susceptibility to malignant hyperthermia, hypokalemic periodic paralysis, and congenital muscle disease (35–37). These studies suggest that these three genes may be involved in heart failure, bacterial infections, muscle wasting, and other types of diseases. Analysis of gene function revealed that *CACNA1S*, *ATP2A3*, and *MYH7* were involved in cardiac muscle contraction and adrenergic signaling in cardiomyocytes, both of which are associated with heart failure and other diseases. Necropsy of dead chickens showed that most of them had the phenomena of swollen liver, spleen, and heart, thickened pericardium, and increased pericardial fluid. We hypothesized that mutations in the exons of *CACNA1S*, *ATP2A3*, and *MYH7* genes are associated with chicken *S. pullorum* infection and may be important candidates for disease resistance/susceptibility. None of these candidate genes has been found in previous studies of *Salmonella* for three possible reasons. To start with, the strains of *S. pullorum* and breeds of chicken used in this study were different from those in previous studies. Moreover, the trait we focused on was the death and survival of chicks infected with *S. pullorum*, which was also different from previous studies. Additionally, this study mined for disease candidate genes using whole-genome resequencing and genome-wide association study, unlike most studies that focus on differences in gene expression after bacterial infection.

### Chicken *S. pullorum* infection and gut microbiota

*Salmonella*, as an intracellular bacterium that can cause local or systemic infection, has important links with economy and public health. It is one of the major pathogens of global food safety, and poultry is its main vector. Antibiotics have been the main strategy for controlling *Salmonella* infection for many years. However, the overuse of antibiotics, the resistance of bacterial pathogens, and the global ban on antibiotics have led to the use of antibiotic alternatives being recommended and successfully applied in many countries. For example, feed- (prebiotics, probiotics, bacterial metabolites, organic acids, essential oils, etc.) and nonfeed-based (phages, nanoparticles, and vaccines) control strategies have been gradually developed in poultry production (38, 39). Among them, adding probiotics or prebiotics to improve the gut microecological balance of poultry is the greatest concern of researchers. A number of studies have demonstrated the important potential of prebiotics and probiotics in reducing the incidence of *Salmonella* and its adverse effects on the gastrointestinal tract of poultry (40–44). Therefore, it is crucial to understand the gut microbial characteristics of *Salmonella*-infected chickens, which will help us choose appropriate intervention or treatment measures to restore the homeostasis of gut microecology, so as to inhibit the proliferation of *Salmonella* in the chicken.

The analysis of differential bacteria in this study showed that *Lactobacillus* and *Escherichia_Shigella* were significantly higher in the ND group than in the NS group, and they are also important marker bacteria genera of NS. Studies have shown that *Lactobacillus* can reduce the colonization of pathogens in the host by stimulating the adaptive immunity, changing the cecal microbial structure, and producing inhibitory metabolites, such as organic acid (45). For example, the addition of *Lactobacillus* in feed can enhance the infection resistance of broilers to *S. pullorum* (46) and inhibit the production of multi-drug resistant *S, enteritidis* in eggs (47). In our study, we also found that *S. pullorum* infection in chicks significantly reduced the abundance of *Lactobacillus* compared with the unchallenged group, suggesting that the bacterium may play an important role in the fight against *S. pullorum* infection. *Escherichia_Shigella* belongs to Proteobacteria and is generally considered to be nonpathogenic bacteria. However, when stimulated by stress, *Escherichia_Shigella* can become pathogenic bacteria, leading to the occurrence of disease (48). It can enter the intestinal mucosa through M cells and then cause intestinal inflammation or animal diarrhea with high morbidity and mortality. Studies on Tibetan pig gut microbiota have found that *Escherichia_Shigella* is significantly correlated with diseases, such as yellow and white dysentery (49). Moreover, *Escherichia_Shigella*, *Escherichia coli*, and *Salmonella* have the same target cell in the intestinal infection process (50). In our study, chicks infected with *S. pullorum* also showed dysentery or anal paste phenomenon, even dying from anal paste. The abundance of *Escherichia_Shigella* in the cecum of chicks in the death group was significantly increased. These further hints that *Escherichia_Shigella* may be the signature genus of *S. pullorum* infection and chick death. The abundance of *Bacillus* was significantly higher in the survival group than that in the death group, and it also was a marker genus in this group. The addition of *Bacillus* in the diet can improve the immune response of *S. enteritidis*-infected broilers (51), regulate the gut microbial imbalance caused by *S. enteritidis* infection (52), and inhibit the proliferation of *S. enteritidis* in laying hens (53). Similarly, *S. typhimurium* infection can also reduce the diversity and abundance of gut microbiota in chickens, and the imbalance of gut microbiota will affect the production of organic acids and vitamins. The addition of *Bacillus* in the diet can also restore the gut microbial imbalance caused by *S. typhimurium* infection (54). Combined with our study, *Lactobacillus*, *Escherichia_Shigella*, and *Bacillus* may be the signature genera associated with *S. pullorum* infection. They may provide microbial markers for the purification of pullorum disease and also provide basic reference for adjusting gut microbial environment through fecal bacteria transplantation, probiotics, and other means to block the transmission of chicken pullorum disease and realize healthy breeding.

## MATERIALS AND METHODS

### Animal and bacterial strain

Xin Pudong chickens were selected from the Animal Husbandry and Veterinary Research Institute of Shanghai Academy of Agricultural Sciences. We selected 23 chickens positive of pullorum disease, including six roosters and 17 hens (29 weeks old), and 98 chickens negative of pullorum disease, including 12 roosters and 86 hens (29 weeks old). Positive roosters were mated with positive hens to obtain 227 positive offspring chicks, and negative roosters were mated with negative hens to obtain 611 negative offspring chicks. All the chickens were maintained at the same condition and were not treated with antibiotics. The standard strain SP1218 of *S. pullorum* from Shanghai Veterinary Research Institute of Chinese Academy of Agricultural Sciences was selected as the attack strain. All animal experiments were conducted in accordance with the Laboratory Animal Research guide of Shanghai Jiao Tong University, China.

### Whole blood plate agglutination test

A pplate agglutination test antigen kit (product code 03.01.001.001; Beijing Zhonghai Biotech Co., Ltd., China) was used to detect the *S. pullorum* antibodies of Xin Pudong chickens. The procedure was as follows: (i) one drop (about 50 µL) of antigen was sucked by the tip of the gun and dropped vertically on the glass plate; (ii) wipe the tip of the chicken’s comb (or the brachial vein under the chicken wing) with an alcohol cotton ball to disinfect; bleed with a sterilized needle; and the blood sample equal to the antigen was collected by pipetting gun; (iii) the blood and antigen were mixed and spread to a liquid surface with a diameter of about 2.0 cm; (iv) the results were determined within 2 min compared with the control; and (v) if 100% (+++) agglutination occurred, it was considered as a strong positive; if 50% (++) agglutination occurred, it was considered as a weak positive. If agglutination does not occur, it was negative.

### Determination of the median lethal dose of *S. pullorum*

The standard strain SP1218 of *S. pullorum* was used as the bacteria for median lethal dose (LD50) determination. Pick a single-colony bacterium on the plate and streak the plate. After 18 h of culture, the bacteria were washed with sterile PBS, and the bacterial solution was collected. After low-speed centrifugation, the bacteria were washed with precooled PBS for three times and finally resuspended in PBS. The OD_600_ value of the bacterial solution was measured. Bacterial suspensions with different gradients were prepared according to OD_600_ values. When OD_600_ = 1, the bacterial solution concentration was 5 × 10^8^ colony-forming units (CFU)/mL, which needs to be concentrated into 10^9^ CFU/mL by low-speed centrifugation. Finally, we collected six different concentrations of bacterial solution, which were 5 × 10^9^, 5 × 10^8^, 5 × 10^7^, 5 × 10^6^, 5 × 10^5^CFU/mL, and 5 × 10^4^ CFU/mL. The above six different concentrations of bacterial suspension were inoculated into the chicks by intramuscular injection in the leg at a dose of 0.2 mL per chick. The modified Käber method was used to calculate LD50 with the following formula:


$$LD50=\log^{-[Xm-i[[\sum p-(3-Pm-Pn)/4]]}.$$

This formula was used when 100 and 0% were not included in the mortality data, where Xm represents the log of the maximum challenge dose at base 10; i represents the log difference of the challenge dose, which is generally 1; ∑*P* represents the sum of mortality; Pm represents maximum mortality; Pn represents the minimum mortality rate.

### Chick was challenged with *S. pullorum*

According to the challenge dose obtained by the LD50 test, the 3 day-old chicks were injected intramuscularly with a suspension of SP1218, 0.2 mL per chick. All chickens were fed *ad libitum* and raised without antibiotics and vaccine. The death and survival of chickens were counted every day, and the mortality and survival rates were calculated. Samples of liver, spleen, and cecal contents were collected and stored in a −80°C refrigerator after the challenge test.

### Detection of the visceral bacterial load

The liver and spleen samples were collected from the challenged chickens, and their grinding liquid was cultured with SS agar medium to calculate the visceral bacteria load. The operation is as follows: 1 g of viscera was homogenized by adding 9 mL normal saline; then, the dilution ratio was 10^0^, 10^1^, 10^2^, 10^3^, 10^4^, 10,^5^ and 10^6^. The dilution ratio of 10^4^ and 10^5^ was suitable for counting. Accordingly, 30 µL solution was coated on SS AGAR medium; each dilution factor was set with three replicates and counted after culture for 29–36 h. The SP1218 was verified by PCR with the primer of SGP special-F: TAGTCATGACAGCGTCCTGTC and SGP special-R: AGTGGCTGACGTACGGCA. The PCR reaction system (20 µL system): 2× Taq PCR Master Mix 10 µL, DNA template 1 µL, forward primers 1 µL, reverse primers 1 µL, and supplement ddH_2_O to 20 µL; PCR reaction parameters: 94°C for 2 min, 35 cycles of 94°C for 50 s, 57°C for 30 s, and 72°C for 50 s, and 72°C for 10 min. 10 µL PCR amplified products were placed on 1.5% agarose gel and underwent 120 V constant-pressure electrophoresis for 30 min to identify the PCR products. The results were observed and recorded under a UV lamp. The target fragments were recycled by the GeneJET Gel Extraction Kit (number K0691, Thermo Scientific, United States) and subjected to Sanger sequencing.

### Whole-genome resequencing

The liver samples were collected, and genomic DNA was extracted for whole-genome resequencing. A total of 149 libraries were constructed with the TruSeqTM DNA Sample Prep Kit (Cat #FC-121–2003, Illumina, United States). Sequencing was carried out by Illumina NovaSeq platform using the paired-end (2 × 150 bp) method in the Shanghai Personal Biotechnology Limited Company (Shanghai, P.R. China).

Data filtration mainly includes joint pollution removal, mass filtration, and length filtration. The sequencing reads were compared with the chicken reference genome GRCg6a in National Center for Biotechnology Information by BWA software (Version 0.7.12-r1039) (55). The genetic variation was then detected using the HaplotypeCaller program of the GenomeAnalysisTK software (Version 4.0) (56). Plink software (Version 1.9) (57) was used to filter the genetic variation. The criteria were as follows: (i) the minor allele frequency should be greater than 0.05; (ii) deleting the loci with genotype deletion rate greater than 5%; and (3) deleting the individuals with loci missing rate greater than 50%.

### Genome-wide association analysis

Based on phenotypic and genotypic data of the death and survival groups, we analyzed genetic variations that were significantly associated with the host susceptibility/resistance-related genes in *S. pullorum*-infected chickens. GEMMA software (version 0.96) (58) was used to perform genome-wide association study (GWAS) based on the univariate linear mixed model:


y=Wα+xβ+u+ε.

Thereinto, *y* represents the characteristic value of dead or survival state, namely, “0” or “1”; *W* is a covariable matrix (i.e., fixed effects) controlling population structure and batch influence; *α* is the vector containing the corresponding coefficient of the intercept; *x* is the vector of the single nucleotide polymorphisms (SNPs) effect; *β* is the effect of SNPs; and *u* is a random polygenic effect vector with covariance structure subject to *u* ~ *N*(0, *KVg*), where *K* is the genomic relationship matrix derived from independent SNPs; *Vg* is the additive variance of polygenic; and *ε* is the vector of random error. The Wald test statistics was used to test the null hypothesis for each SNP (*β* = 0). Principal components analysis, Manhattan, and Q–Q plots were drawn using R (Version 3.6.2) (59). The genome-wide significance threshold was set to less than 0.05/SNPs, and the suggestive significance level was determined by a value of 5/SNPs.

Genetic variation annotation is implemented through ANNOVAR (version 2017) (60). Based on the genetic variation location on the reference genome, it can be distinguished whether the variation locus was distributed in the inter-gene, gene, or CDS region, and the type was nonsynonymous or synonymous mutation. The genes where the significant genetic variants were located (and their adjacent genes) were selected for functional enrichment analysis. Genes were annotated according to Gene Ontology and KEGG databases.

### 16S rRNA gene sequencing

Cecal contents of dead and surviving chicks were collected after the challenge test for gut microbial genome sequencing. The fecal genome DNA extraction kit (Cat #DP328, Tiangen, China) was used to extract the gut microbial DNA. The V3–V4 hypervariable region of the gut microbial 16S rRNA gene was amplified and sequenced using the NovaSeq PE250 platform (Illumina, United States).

The Quantitative Insights Into Microbial Ecology 2 (QIIME2, version 2019.4) pipeline was employed to process the sequencing data, including primer removal, denoise, splice, chimeras deletion, and dereplication. The sequence obtained after quality control is called amplicon sequence variants (ASVs), corresponding to the operational taxonomic units (OTUs). Combine the ASV feature sequence with the ASV table and remove singleton ASVs (i.e., ASVs with only one sequence in the total sample). After the ASV feature sequence is obtained, the length distribution of the high-quality sequences contained in all the samples is counted.

### Species annotation and diversity analysis

The Silva database was used to compare and annotate 16S rRNA genes of bacteria. To minimize the difference in sequencing depth across samples, it is necessary to obtain an averaged, rounded rarefied OTU abundance table. We randomly select a certain number of sequences from each sample, which is the sparse method, to ensure that all samples reach the same depth. Then, the observed OTU and its relative abundance of each sample at this sequencing depth were predicted. For this evenly resampled OTU table, we analyzed and obtained the number of taxa contained in each sample at each taxonomic level (domain, phylum, class, order, family, genus, species). Then, the column chart and heat map were used to show the taxonomic composition of the microbial community.

The alpha of the microbial community was analyzed based on the OTU abundance table. The indexes of Chao1, Shannon, Faith’s PD, Pielou’s evenness, and Good’s coverage represent the microbial community’s richness, diversity, evolution-based diversity, evenness, and coverage, respectively. The differences in gut microbial beta diversity can be displayed by NMDS analysis and PCoA. The heat map shows the differences in the microbial composition between groups. Only the top 20 genera are listed. Two-sided *t*-tests were applied to identify different taxa microbes among groups. PICRUSt2 was used to predict the composition of microbial genes or functional units based on the 16S rRNA sequencing data, so as to understand the potential function of the microbial community.

## Data Availability

Raw read sequences are publicly available in the Sequence Read Archive at National Center for Biotechnology Information (NCBI) under the BioProject accession numbers PRJNA1042676 and PRJNA1042663.

## SUPPLEMENTAL MATERIAL

Supplemental figures and tables spectrum.00392-24-s0001.docx Figures S1 to S5; Tables S1 to S11.

ASM does not own the copyrights to Supplemental Material that may be linked to, or accessed through, an article. The authors have granted ASM a non-exclusive, world-wide license to publish the Supplemental Material files. Please contact the corresponding author directly for reuse.
