# Supplementary material for: The host susceptibility/resistance-related genes and gut microbial characteristics in Salmonella pullorum-infected chickens
Source: Microbiol Spectr. 2025 Mar 3;13(4):e00392-24. doi: 10.1128/spectrum.00392-24 (PMC11960056; doi:10.1128/spectrum.00392-24)
Supplement: Supplemental figures and tables — Figures S1 to S5; Tables S1 to S11. [file spectrum.00392-24-s0001.docx]

**The host susceptibility/resistance** **related genes and gut microbial** **characteristics in *Salmonella pullorum* infected chickens**

Jinmei Ding^1^, Jianshen Zhu^1^, Hao zhou^1^, Kaixuan Yang^2^, Chao Qin^1^, Yaodong Zhang^3^, Chengxiao Han^1^, Lingyu Yang^1^, Chuan He^1^, Ke Xu^1^, Yuming Zheng^1^, Huaixi Luo^1^, Kangchun Chen^1^, Wenchuan Zhou^1^, Shengyao Jiang^1^, Jiajia Liu^1^, Wenqi Zhu^1^, Qing Niu^2^, Zhenxiang Zhou^2^, Shaohui Wang^3^, Shengqing Yu^3, *^, Qizhong Huang^2, *^, He Meng^1, *^

1 Shanghai Key Laboratory of Veterinary Biotechnology, Department of Animal Science, School of Agriculture and Biology, Shanghai Jiao Tong University, Shanghai, People’s Republic of China

2 Animal Husbandry and Veterinary Research Institute, Shanghai Academy of Agricultural Science, Shanghai, People’s Republic of China

3 Shanghai Veterinary Research Institute, the Chinese Academy of Agricultural Sciences, Shanghai, People’s Republic of China

* Address correspondence to Shengqing Yu, yus@shvri.ac.cn. Qizhong Huang, [huangqizh@163.com](mailto:huangqizh@163.com). He Meng, menghe@sjtu.edu.cn

**Supplementary figures and tables**


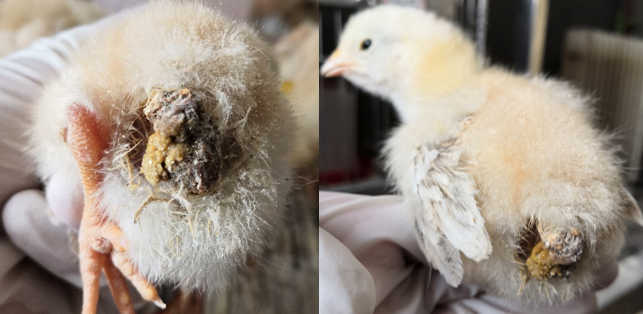


Supplementary figure 1 The chicks appeared paste anal phenomenon


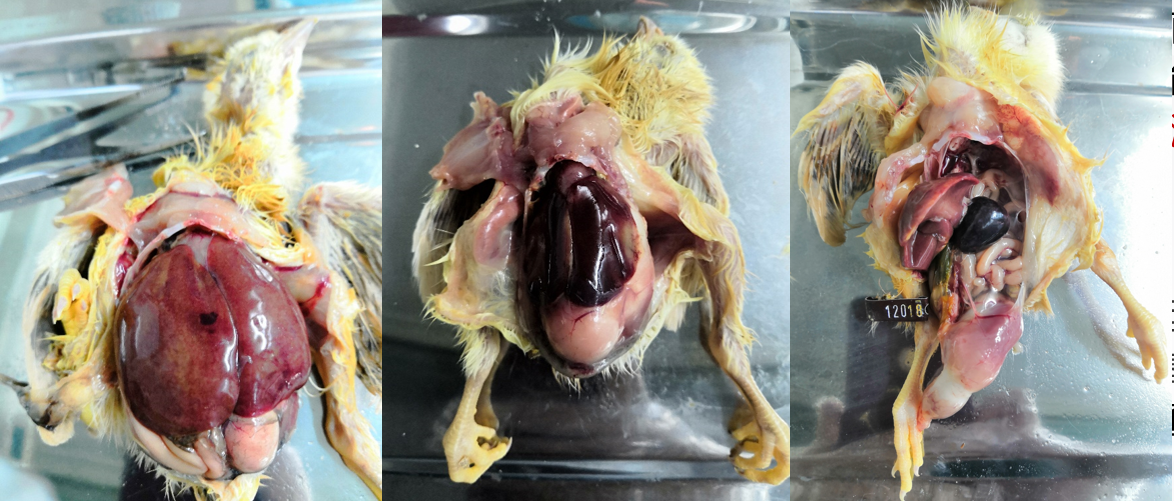


Supplementary figure 2 Liver and spleen lesions in deceased individuals


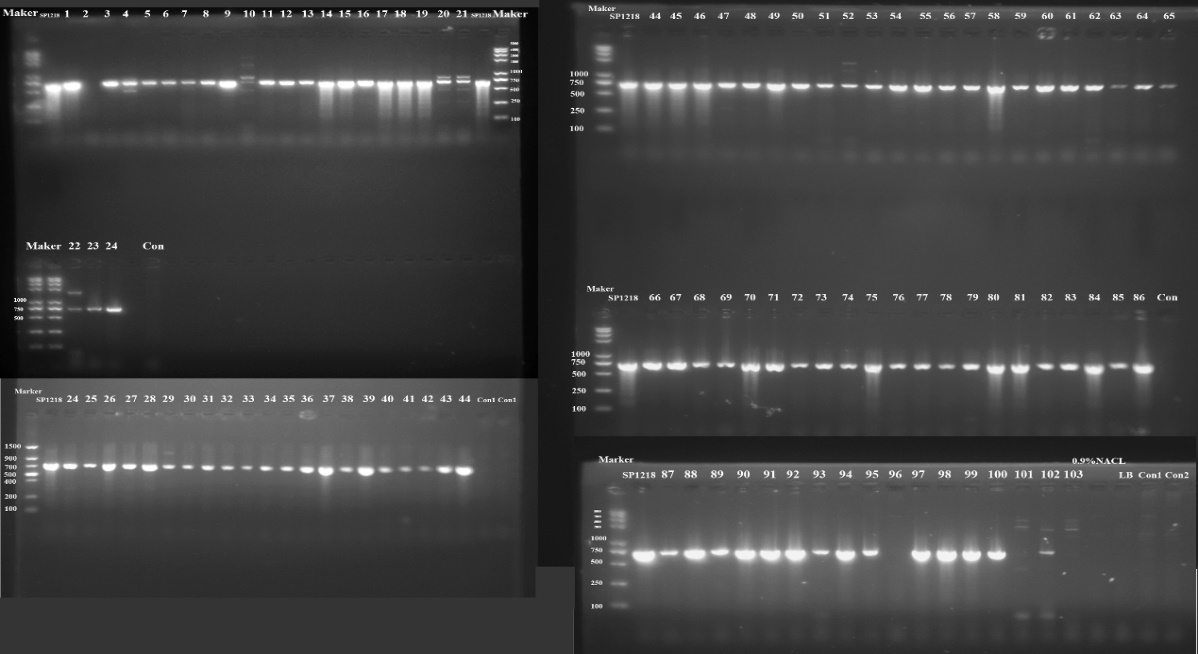


Supplementary figure 3 *Salmonella* *pullorum* (SP1218) was verified by PCR


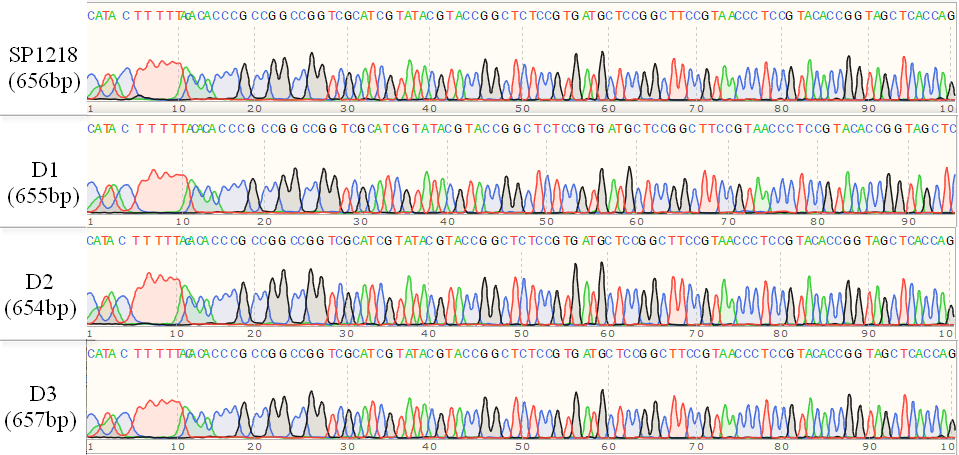


Supplementary figure 4 Comparison of the three samples with standard strain SP1218

Supplementary Table 1 Determination of median lethal dose

| Group | concentration of bacteria (CFU/mL) | Number of chicks | Time and number of dead chickens after challenge | | | | | | |
| --- | --- | --- | --- | --- | --- | --- | --- | --- | --- |
|  |  |  | 1dpi | 2dpi | 3dpi | 4dpi | 5dpi | 6dpi | 7dpi |
| positive offspring chicks | 5x10^4^ | 10 | 0 | 1 | 0 | 0 | 0 | 0 | 0 |
|  | 5x10^5^ | 10 | 0 | 0 | 0 | 1 | 0 | 0 | 0 |
|  | 5x10^6^ | 10 | 0 | 1 | 0 | 1 | 0 | 0 | 0 |
|  | 5x10^7^ | 10 | 0 | 0 | 1 | 2 | 2 | 1 | 0 |
|  | 5x10^8^ | 10 | 0 | 2 | 3 | 1 | 4 | 0 | 0 |
|  | 5x10^9^ | 10 | 1 | 8 | 1 | 0 | 0 | 0 | 0 |
| negative offspring chicks | 5x10^4^ | 10 | 0 | 0 | 0 | 0 | 0 | 0 | 0 |
|  | 5x10^5^ | 10 | 0 | 0 | 0 | 0 | 0 | 0 | 0 |
|  | 5x10^6^ | 10 | 0 | 0 | 1 | 0 | 0 | 0 | 0 |
|  | 5x10^7^ | 10 | 0 | 0 | 0 | 2 | 2 | 2 | 0 |
|  | 5x10^8^ | 10 | 0 | 3 | 2 | 2 | 0 | 0 | 0 |
|  | 5x10^9^ | 10 | 0 | 8 | 2 | 0 | 0 | 0 | 0 |

Supplementary table 2 Comparison of three samples with standard strain of SP1218

| Sample | High Scores | Practical Score | Sequencing coverage (%) | Consistency (%) | Standard Strain |
| --- | --- | --- | --- | --- | --- |
| D1 | 1179 | 1179 | 99 | 99.24 | SP1218 |
| D2 | 1170 | 1170 | 96 | 100 | SP1218 |
| D3 | 1170 | 1170 | 96 | 100 | SP1218 |

Supplementary table 3 SNPs annotated to genes significantly associated with *Salmonella pullorum* infection

| Chr | position | ref/alt | p value | genes | Mutating location |
| --- | --- | --- | --- | --- | --- |
| 1 | 125067 | A/G | 7.89E-09 | DHRS4(dist=53457), CLC2DL3(dist=176368) | intergenic |
| 1 | 135555 | A/G | 6.32E-07 | DHRS4(dist=63945), CLC2DL3(dist=165880) | intergenic |
| 1 | 135889 | C/T | 3.71E-08 | DHRS4(dist=64279), CLC2DL3(dist=165546) | intergenic |
| 1 | 135904 | A/G | 8.09E-08 | DHRS4(dist=64294), CLC2DL3(dist=165531) | intergenic |
| 1 | 40208052 | G/A | 6.34E-07 | MYF5(dist=121726), NTS(dist=1999501) | intergenic |
| 1 | 40210084 | G/T | 1.22E-06 | MYF5(dist=123758), NTS(dist=1997469) | intergenic |
| 1 | 45878705 | T/C | 1.01E-06 | ELK3(dist=63116), NEDD1(dist=163829) | intergenic |
| 1 | 45955085 | C/T | 9.76E-07 | ELK3(dist=139496), NEDD1(dist=87449) | intergenic |
| 1 | 90840932 | G/A | 2.45E-09 | EPHA3(dist=205469), ARL6(dist=1795641) | intergenic |
| 1 | 90841070 | C/A | 2.77E-07 | EPHA3(dist=205607), ARL6(dist=1795503) | intergenic |
| 1 | 90919038 | G/C | 4.93E-07 | EPHA3(dist=283575), ARL6(dist=1717535) | intergenic |
| 1 | 90919100 | A/G | 6.19E-07 | EPHA3(dist=283637), ARL6(dist=1717473) | intergenic |
| 1 | 96568933 | G/A | 6.59E-08 | MIR1806(dist=1334557) | intergenic |
| 1 | 96568937 | A/T | 8.50E-08 | MIR1806(dist=1334561) | intergenic |
| 1 | 96568968 | T/C | 2.47E-07 | MIR1806(dist=1334592) | intergenic |
| 1 | 96570092 | G/A | 5.56E-07 | MIR1806(dist=1335716) | intergenic |
| 1 | 96570119 | G/A | 8.25E-07 | MIR1806(dist=1335743) | intergenic |
| 1 | 112690643 | A/G | 1.45E-08 | MAOA(dist=42854), GPR34(dist=509442) | intergenic |
| 1 | 112693277 | C/G | 1.23E-08 | MAOA(dist=45488), GPR34(dist=506808) | intergenic |
| 1 | 113073693 | C/T | 7.69E-07 | MAOA(dist=425904), GPR34(dist=126392) | intergenic |
| 1 | 185063016 | T/C | 7.27E-08 | CNTN5 | intronic |
| 2 | 39270 | A/G | 3.07E-07 | ABCF2(dist=120335) | intergenic |
| 2 | 39271 | T/C | 3.07E-07 | ABCF2(dist=120334) | intergenic |
| 2 | 39518 | G/A | 9.93E-07 | ABCF2(dist=120087) | intergenic |
| 3 | 51806265 | C/T | 1.43E-12 | TIAM2(dist=1128167), GTF2H5(dist=158342) | intergenic |
| 3 | 51806274 | A/C | 3.47E-11 | TIAM2(dist=1128176), GTF2H5(dist=158333) | intergenic |
| 3 | 51806310 | A/G | 2.69E-17 | TIAM2(dist=1128212), GTF2H5(dist=158297) | intergenic |
| 3 | 51806342 | A/G | 3.97E-14 | TIAM2(dist=1128244), GTF2H5(dist=158265) | intergenic |
| 3 | 51806368 | C/T | 7.81E-11 | TIAM2(dist=1128270), GTF2H5(dist=158239) | intergenic |
| 3 | 51806396 | C/A | 5.92E-10 | TIAM2(dist=1128298), GTF2H5(dist=158211) | intergenic |
| 3 | 95279509 | G/A | 1.03E-06 | RSAD2(dist=282774), ID2(dist=574430) | intergenic |
| 4 | 90708600 | G/A | 1.68E-11 | GNOT2(dist=978680), LBX2(dist=224415) | intergenic |
| 4 | 90708634 | C/T | 2.19E-11 | GNOT2(dist=978714), LBX2(dist=224381) | intergenic |
| 6 | 1153005 | G/T | 8.48E-07 | MIR7478(dist=48892) | intergenic |
| 6 | 1153033 | A/C | 1.50E-07 | MIR7478(dist=48920) | intergenic |
| 6 | 1154572 | G/C | 3.52E-08 | MIR7478(dist=50459) | intergenic |
| 6 | 1156834 | G/A | 1.06E-06 | MIR7478(dist=52721) | intergenic |
| 6 | 1159606 | A/T | 8.98E-07 | MIR7478(dist=55493) | intergenic |
| 6 | 1181342 | C/A | 3.02E-09 | MIR7478(dist=35533) | intergenic |
| 6 | 10545254 | G/C | 4.77E-07 | SGMS1(dist=94406), MIR1772(dist=625450) | intergenic |
| 7 | 39785 | T/C | 1.70E-07 | NAB1(dist=54092) | intergenic |
| 7 | 39794 | T/C | 7.78E-07 | NAB1(dist=54083) | intergenic |
| 7 | 39839 | G/A | 7.07E-07 | NAB1(dist=54038) | intergenic |
| 7 | 39841 | A/G | 2.70E-07 | NAB1(dist=54036) | intergenic |
| 7 | 39844 | T/C | 6.62E-07 | NAB1(dist=54033) | intergenic |
| 7 | 39903 | C/T | 1.10E-06 | NAB1(dist=53974) | intergenic |
| 7 | 39906 | T/A | 5.78E-07 | NAB1(dist=53971) | intergenic |
| 9 | 5036551 | C/G | 1.17E-06 | ATG4B(dist=63304), MIR1704(dist=23194) | intergenic |
| 10 | 15141538 | C/T | 7.50E-07 | RGMA(dist=10525), NR2F2(dist=1129065) | intergenic |
| 10 | 15141540 | C/T | 1.09E-06 | RGMA(dist=10527), NR2F2(dist=1129063) | intergenic |
| 11 | 20013801 | A/G | 2.78E-07 | CHST6(dist=87269) | intergenic |
| 11 | 20016948 | C/G | 2.86E-08 | CHST6(dist=90416) | intergenic |
| 12 | 209600 | G/T | 7.03E-08 | DUSP7(dist=432355) | intergenic |
| 12 | 209623 | T/C | 1.72E-07 | DUSP7(dist=432332) | intergenic |
| 12 | 250345 | A/T | 1.29E-08 | DUSP7(dist=391610) | intergenic |
| 13 | 2339417 | A/G | 3.49E-07 | SRA1(dist=30508), PCDHGA2(dist=204590) | intergenic |
| 14 | 228316 | A/C | 1.34E-07 | RRN3(dist=258538) | intergenic |
| 14 | 228321 | A/C | 3.94E-08 | RRN3(dist=258533) | intergenic |
| 14 | 307695 | A/G | 8.33E-07 | RRN3(dist=179159) | intergenic |
| 14 | 307755 | A/G | 7.66E-07 | RRN3(dist=179099) | intergenic |
| 14 | 311094 | T/G | 1.13E-08 | RRN3(dist=175760) | intergenic |
| 14 | 311098 | A/T | 2.50E-09 | RRN3(dist=175756) | intergenic |
| 14 | 311296 | C/T | 8.59E-07 | RRN3(dist=175558) | intergenic |
| 14 | 311372 | G/C | 1.56E-09 | RRN3(dist=175482) | intergenic |
| 14 | 340154 | G/A | 2.98E-12 | RRN3(dist=146700) | intergenic |
| 14 | 340301 | A/T | 4.13E-08 | RRN3(dist=146553) | intergenic |
| 14 | 344414 | T/C | 1.34E-06 | RRN3(dist=142440) | intergenic |
| 14 | 349498 | G/A | 4.42E-10 | RRN3(dist=137356) | intergenic |
| 14 | 350313 | A/T | 9.16E-08 | RRN3(dist=136541) | intergenic |
| 14 | 350317 | A/G | 2.49E-08 | RRN3(dist=136537) | intergenic |
| 14 | 350319 | T/G | 1.47E-08 | RRN3(dist=136535) | intergenic |
| 14 | 397675 | G/C | 1.47E-08 | RRN3(dist=89179) | intergenic |
| 14 | 398098 | G/A | 5.30E-07 | RRN3(dist=88756) | intergenic |
| 14 | 399056 | T/G | 1.94E-11 | RRN3(dist=87798) | intergenic |
| 14 | 402107 | C/A | 9.18E-07 | RRN3(dist=84747) | intergenic |
| 14 | 412254 | C/A | 1.29E-09 | RRN3(dist=74600) | intergenic |
| 14 | 412285 | C/T | 2.92E-15 | RRN3(dist=74569) | intergenic |
| 15 | 278779 | G/T | 2.47E-09 | SNAP29(dist=99793) | intergenic |
| 15 | 13017597 | C/A | 1.53E-08 | SDSL(dist=151174) | intergenic |
| 16 | 1250609 | A/T | 1.21E-06 | MIR7480-1(dist=152727) | intergenic |
| 18 | 11033965 | T/A | 2.81E-09 | GRB2(dist=258520) | intergenic |
| 18 | 11155795 | C/T | 8.84E-07 | GRB2(dist=380350) | intergenic |
| 19 | 58530 | T/G | 4.58E-07 | MYH7 | exonic |
| 19 | 100508 | T/C | 5.56E-07 | MYH7(dist=29513), BUD23(dist=181205) | intergenic |
| 19 | 292512 | T/C | 5.85E-07 | BUD23(dist=6049), MLXIPL(dist=73366) | intergenic |
| 19 | 292522 | A/G | 5.08E-07 | BUD23(dist=6059), MLXIPL(dist=73356) | intergenic |
| 19 | 292549 | T/C | 6.63E-09 | BUD23(dist=6086), MLXIPL(dist=73329) | intergenic |
| 19 | 292552 | G/A | 6.63E-09 | BUD23(dist=6089), MLXIPL(dist=73326) | intergenic |
| 19 | 292561 | A/G | 1.08E-08 | BUD23(dist=6098), MLXIPL(dist=73317) | intergenic |
| 19 | 292562 | C/T | 1.08E-08 | BUD23(dist=6099), MLXIPL(dist=73316) | intergenic |
| 19 | 292564 | G/A | 6.63E-09 | BUD23(dist=6101), MLXIPL(dist=73314) | intergenic |
| 19 | 292567 | T/C | 6.63E-09 | BUD23(dist=6104), MLXIPL(dist=73311) | intergenic |
| 19 | 292588 | A/G | 8.24E-09 | BUD23(dist=6125), MLXIPL(dist=73290) | intergenic |
| 19 | 3439993 | T/C | 1.19E-06 | ATP2A3 | exonic |
| 19 | 3440003 | A/C | 1.19E-06 | ATP2A3 | exonic |
| 19 | 3440011 | G/C | 1.07E-06 | ATP2A3 | exonic |
| 19 | 3440026 | A/G | 1.80E-07 | ATP2A3 | exonic |
| 19 | 3440027 | C/T | 1.80E-07 | ATP2A3 | exonic |
| 19 | 3440032 | G/C | 2.53E-07 | ATP2A3 | exonic |
| 19 | 3440041 | T/C | 4.45E-07 | ATP2A3 | exonic |
| 19 | 3440053 | T/C | 2.35E-07 | ATP2A3 | exonic |
| 19 | 3440056 | C/T | 4.04E-07 | ATP2A3 | exonic |
| 19 | 3440065 | C/G | 2.50E-07 | ATP2A3 | exonic |
| 19 | 3445965 | G/A | 1.28E-06 | ATP2A3 | exonic |
| 19 | 3449102 | T/G | 8.40E-07 | ATP2A3 | exonic |
| 20 | 19014 | T/C | 7.33E-08 | IGSF1(dist=53202) | intergenic |
| 20 | 19772 | G/T | 1.16E-07 | IGSF1(dist=52444) | intergenic |
| 20 | 19773 | A/T | 1.16E-07 | IGSF1(dist=52443) | intergenic |
| 20 | 20227 | T/G | 1.07E-07 | IGSF1(dist=51989) | intergenic |
| 20 | 23948 | C/T | 1.37E-09 | IGSF1(dist=48268) | intergenic |
| 20 | 24866 | C/T | 6.05E-08 | IGSF1(dist=47350) | intergenic |
| 20 | 34774 | G/T | 5.64E-13 | IGSF1(dist=37442) | intergenic |
| 20 | 38497 | C/T | 1.23E-07 | IGSF1(dist=33719) | intergenic |
| 22 | 119902 | G/A | 7.96E-07 | MIR6576(dist=78096) | intergenic |
| 22 | 120214 | A/T | 9.80E-07 | MIR6576(dist=77784) | intergenic |
| 22 | 5376078 | A/G | 5.49E-08 | CASP14(dist=56504) | intergenic |
| 22 | 5388608 | T/G | 2.92E-07 | CASP14(dist=69034) | intergenic |
| 22 | 5399788 | G/A | 1.44E-07 | CASP14(dist=80214) | intergenic |
| 22 | 5399796 | A/T | 5.28E-08 | CASP14(dist=80222) | intergenic |
| 22 | 5399834 | A/G | 9.09E-07 | CASP14(dist=80260) | intergenic |
| 23 | 7619 | C/T | 1.46E-09 | MIR7478(dist=26230) | intergenic |
| 23 | 11861 | G/A | 7.31E-09 | MIR7478(dist=21988) | intergenic |
| 23 | 55292 | T/G | 1.01E-07 | MIR7478(dist=21366), RPS6KA1L(dist=173600) | intergenic |
| 24 | 3621523 | G/T | 2.58E-07 | TECTA (dist=33729), TMEM136(dist=191227) | intergenic |
| 24 | 6395178 | A/T | 2.03E-07 | CRYAB(dist=148943) | intergenic |
| 24 | 6395191 | C/T | 1.54E-07 | CRYAB(dist=148956) | intergenic |
| 25 | 563863 | G/A | 2.46E-11 | S100A10(dist=97151), ANP32E(dist=76595) | intergenic |
| 25 | 2123673 | G/C | 3.05E-07 | UBQLN4(dist=573188), LOC100859191(dist=26448) | intergenic |
| 25 | 2137574 | C/A | 3.26E-07 | UBQLN4(dist=587089), LOC100859191(dist=12547) | intergenic |
| 25 | 2139225 | A/G | 5.01E-09 | UBQLN4(dist=588740), LOC100859191(dist=10896) | intergenic |
| 25 | 2139237 | A/C | 3.15E-07 | UBQLN4(dist=588752), LOC100859191(dist=10884) | intergenic |
| 25 | 2141984 | T/G | 9.70E-07 | UBQLN4(dist=591499), LOC100859191(dist=8137) | intergenic |
| 25 | 3083201 | A/G | 8.91E-08 | CKS1B(dist=61590), LOC769121(dist=36713) | intergenic |
| 25 | 3085069 | A/G | 8.04E-09 | CKS1B(dist=63458), LOC769121(dist=34845) | intergenic |
| 25 | 3085748 | G/A | 3.23E-07 | CKS1B(dist=64137), LOC769121(dist=34166) | intergenic |
| 25 | 3088015 | G/A | 1.43E-07 | CKS1B(dist=66404), LOC769121(dist=31899) | intergenic |
| 25 | 3088135 | G/C | 9.61E-08 | CKS1B(dist=66524), LOC769121(dist=31779) | intergenic |
| 26 | 275357 | T/C | 8.33E-07 | CATSPER2 | exonic |
| 26 | 276574 | T/C | 8.83E-07 | CATSPER2 | exonic |
| 26 | 276599 | G/T | 2.86E-07 | CATSPER2 | exonic |
| 26 | 276601 | G/C | 2.86E-07 | CATSPER2 | exonic |
| 26 | 276604 | G/C | 1.79E-07 | CATSPER2 | exonic |
| 26 | 276614 | A/G | 4.52E-07 | CATSPER2 | exonic |
| 26 | 276631 | T/C | 1.15E-06 | CATSPER2 | exonic |
| 27 | 187628 | A/G | 4.18E-07 | - | intergenic |
| 27 | 238469 | G/A | 5.89E-07 | - | intergenic |
| 27 | 486423 | A/G | 1.25E-06 | DAD1(dist=1789588) | intergenic |
| 27 | 517515 | C/G | 7.29E-08 | DAD1(dist=1758496) | intergenic |
| 27 | 1540374 | C/G | 5.05E-07 | DAD1(dist=735637) | intergenic |
| 27 | 1571595 | T/C | 7.90E-08 | DAD1(dist=704416) | intergenic |
| 27 | 1684011 | T/A | 9.85E-07 | DAD1(dist=592000) | intergenic |
| 27 | 1706014 | G/C | 9.99E-07 | DAD1(dist=569997) | intergenic |
| 27 | 1706221 | A/C | 3.21E-07 | DAD1(dist=569790) | intergenic |
| 27 | 1706225 | A/T | 2.86E-07 | DAD1(dist=569786) | intergenic |
| 27 | 1708279 | T/C | 5.46E-10 | DAD1(dist=567732) | intergenic |
| 27 | 1710211 | C/A | 1.31E-08 | DAD1(dist=565800) | intergenic |
| 27 | 1788135 | T/A | 3.44E-08 | DAD1(dist=487876) | intergenic |
| 27 | 1788137 | G/A | 2.24E-08 | DAD1(dist=487874) | intergenic |
| 27 | 1788138 | A/C | 3.44E-08 | DAD1(dist=487873) | intergenic |
| 27 | 1788139 | G/A | 2.24E-08 | DAD1(dist=487872) | intergenic |
| 28 | 5066753 | G/T | 4.36E-07 | TNFAIP8L1(dist=19519) | intergenic |
| 31 | 50647 | C/A | 8.56E-09 | - | intergenic |
| 31 | 50778 | G/A | 1.31E-06 | - | intergenic |
| 31 | 190424 | G/C | 7.22E-09 | - | intergenic |
| 31 | 190950 | G/T | 9.84E-09 | - | intergenic |
| 31 | 191713 | T/A | 1.14E-06 | - | intergenic |
| 31 | 191728 | G/C | 4.65E-08 | - | intergenic |
| 31 | 199927 | G/A | 4.05E-08 | - | intergenic |
| 31 | 201490 | A/G | 2.85E-07 | - | intergenic |
| 31 | 201506 | T/G | 4.30E-08 | - | intergenic |
| 31 | 203405 | T/C | 1.56E-08 | - | intergenic |
| 31 | 205379 | G/A | 1.27E-11 | - | intergenic |
| 31 | 206565 | G/A | 2.79E-08 | - | intergenic |
| 31 | 206597 | G/T | 9.00E-07 | - | intergenic |
| 31 | 207291 | T/G | 1.14E-13 | - | intergenic |
| 31 | 215142 | T/G | 1.71E-11 | - | intergenic |
| 31 | 215143 | C/T | 1.67E-10 | - | intergenic |
| 31 | 2115957 | T/G | 1.14E-07 | APEX1(dist=510647) | intergenic |
| 31 | 4534084 | T/C | 2.05E-09 | CHIR-A2(dist=85126), PIT54(dist=388568) | intergenic |
| 31 | 4534296 | T/G | 2.40E-09 | CHIR-A2(dist=85338), PIT54(dist=388356) | intergenic |
| 31 | 4534334 | C/A | 1.65E-09 | CHIR-A2(dist=85376), PIT54(dist=388318) | intergenic |
| 31 | 4535392 | G/T | 2.19E-07 | CHIR-A2(dist=86434), PIT54(dist=387260) | intergenic |
| 31 | 4540692 | T/C | 6.07E-08 | CHIR-A2(dist=91734), PIT54(dist=381960) | intergenic |
| 31 | 4636000 | A/C | 1.54E-07 | CHIR-A2(dist=187042), PIT54(dist=286652) | intergenic |
| 31 | 4646451 | G/T | 1.19E-06 | CHIR-A2(dist=197493), PIT54(dist=276201) | intergenic |
| 31 | 4646455 | A/G | 5.03E-07 | CHIR-A2(dist=197497), PIT54(dist=276197) | intergenic |
| 31 | 4652849 | G/A | 4.66E-07 | CHIR-A2(dist=203891), PIT54(dist=269803) | intergenic |
| 31 | 4662960 | A/G | 1.84E-08 | CHIR-A2(dist=214002), PIT54(dist=259692) | intergenic |
| 33 | 11964 | G/A | 2.98E-11 | - | intergenic |
| 33 | 12006 | A/G | 1.70E-08 | - | intergenic |
| 33 | 12036 | C/A | 5.70E-08 | - | intergenic |
| 33 | 12113 | G/A | 1.86E-08 | - | intergenic |
| 33 | 2994742 | C/T | 3.66E-08 | GR42L6(dist=640957) | intergenic |
| 33 | 3531614 | G/A | 4.72E-16 | GR42L6(dist=104085) | intergenic |
| 33 | 3531645 | T/G | 1.71E-16 | GR42L6(dist=104054) | intergenic |

Supplementary table 4 The InDel and its nearby genes which significantly associated with white Salmonella pullorum infection in chickens

| Chr | start | end | ref/alt | nearby genes | p value |
| --- | --- | --- | --- | --- | --- |
| 1 | 125968 | 125969 | GA/G | DHRS4(dist=54358), CLC2DL3(dist=175466) | 5.11E-07 |
| 1 | 125970 | 125970 | C/CATTCTTGG | DHRS4(dist=54360), CLC2DL3(dist=175465) | 5.11E-07 |
| 1 | 90740878 | 90740880 | CAT/C | EPHA3(dist=105415), ARL6(dist=1895693) | 1.69E-09 |
| 1 | 90740990 | 90740990 | A/AC | EPHA3(dist=105527), ARL6(dist=1895583) | 3.13E-06 |
| 1 | 90841993 | 90841998 | ACAAGG/A | EPHA3(dist=206530), ARL6(dist=1794575) | 2.23E-06 |
| 1 | 90888840 | 90888842 | AAG/A | EPHA3(dist=253377), ARL6(dist=1747731) | 1.38E-06 |
| 1 | 96570087 | 96570087 | C/CA | MIR1806(dist=1335711) | 6.44E-07 |
| 1 | 192664762 | 192664762 | A/AT | HIKESHI(dist=882965), TENM4(dist=543425) | 4.79E-06 |
| 1 | 195581247 | 195581247 | T/TAG | ART7C(dist=524) (upstream) | 5.30E-06 |
| 2 | 80822412 | 80822414 | CCT/C | IKZF1(dist=93218), GRB10(dist=30126) | 4.05E-06 |
| 2 | 148922245 | 148922245 | A/AGTT | FK1L(dist=12916) | 8.10E-08 |
| 3 | 11882630 | 11882632 | ACT/A | PPP3R1(intronic) | 7.65E-06 |
| 3 | 31492722 | 31492732 | TCTCTTGCAGC/T | TTC27(intronic) | 4.78E-06 |
| 4 | 51813117 | 51813121 | CTACA/C | SULT1B1(dist=18225), SPRY1(dist=1544780) | 7.98E-06 |
| 4 | 90745856 | 90745858 | TCA/T | GNOT2(dist=1015936), LBX2(dist=187157) | 2.48E-06 |
| 6 | 1154429 | 1154432 | TAGG/T | MIR7478(dist=50316) | 4.57E-06 |
| 6 | 1194815 | 1194816 | TC/T | MIR7478(dist=22059) | 3.43E-08 |
| 6 | 20952495 | 20952495 | T/TC | MIR7478(dist=66097), DD1CR(dist=56632) | 6.80E-07 |
| 7 | 39775 | 39775 | T/TCGGTC | NAB1(dist=54102) | 8.14E-07 |
| 7 | 39908 | 39909 | TC/T | NAB1(dist=53968) | 2.67E-07 |
| 7 | 39911 | 39911 | G/GA | NAB1(dist=53966) | 1.27E-06 |
| 7 | 39920 | 39921 | TA/T | NAB1(dist=53956) | 8.93E-06 |
| 10 | 560463 | 560463 | C/CA | MIR7478(dist=49241) | 2.80E-07 |
| 10 | 596628 | 596629 | TC/T | MIR7478(dist=85406) | 6.25E-12 |
| 11 | 38638 | 38649 | CCAGCAGTGACA/C | PRMT7(dist=232841) | 1.30E-08 |
| 11 | 54559 | 54559 | C/CACT | PRMT7(dist=216931) | 5.99E-06 |
| 11 | 20017008 | 20017010 | CAA/C | CHST6(dist=90476) | 1.09E-07 |
| 14 | 142539 | 142540 | CA/C | RRN3(dist=344314) | 5.59E-09 |
| 14 | 155710 | 155710 | C/CT | RRN3(dist=331144) | 8.42E-07 |
| 14 | 177576 | 177576 | G/GC | RRN3(dist=309278) | 3.55E-06 |
| 14 | 228310 | 228315 | CGAGGG/C | RRN3(dist=258539) | 3.69E-08 |
| 14 | 228326 | 228331 | GATGCA/G | RRN3(dist=258523) | 2.62E-07 |
| 14 | 307893 | 307894 | CT/C | RRN3(dist=178960) | 1.56E-06 |
| 14 | 340430 | 340441 | ACGCGTGATTTT/A | RRN3(dist=146413) | 6.28E-06 |
| 14 | 350361 | 350362 | TC/T | RRN3(dist=136492) | 3.02E-06 |
| 14 | 402226 | 402231 | AAGGCC/A | RRN3(dist=84623) | 2.68E-06 |
| 14 | 402253 | 402253 | T/TGCAAGGCAAA | RRN3(dist=84601) | 1.41E-09 |
| 14 | 412278 | 412279 | GC/G | RRN3(dist=74575) | 7.27E-07 |
| 15 | 87822 | 87822 | G/GT | SNAP29(dist=290750) | 8.34E-09 |
| 15 | 278825 | 278825 | T/TCAAA | SNAP29(dist=99747) | 1.69E-08 |
| 15 | 13017651 | 13017690 | ACTTGCAAAGAGAAAACATGTCATTTTCACTTTAAATGGG/A | SDSL(dist=151228) | 3.98E-08 |
| 16 | 904646 | 904649 | GACC/G | LOC431499(dist=49986), MIR7480-1(dist=193178) | 3.93E-06 |
| 18 | 10997684 | 10997686 | TAA/T | GRB2(dist=222239) | 1.23E-08 |
| 18 | 11154013 | 11154048 | AGGTTAGGGTTAGGTTAAGGTTGCGATTAGGGTTAG/A | GRB2(dist=378568) | 4.65E-12 |
| 18 | 11155723 | 11155747 | GGTTAGGGTTAGCGTTTAGCTTACT/G | GRB2(dist=380278) | 8.85E-06 |
| 19 | 100389 | 100389 | T/TCCTTGCCTTA | MYH7(dist=29394), BUD23(dist=181324) | 9.77E-06 |
| 19 | 292539 | 292539 | T/TC | BUD23(dist=6076), MLXIPL(dist=73339) | 9.03E-09 |
| 19 | 292542 | 292543 | TC/T | BUD23(dist=6079), MLXIPL(dist=73335) | 9.03E-09 |
| 20 | 5510520 | 5510538 | CTGGTGAGATGAGGGTACA/C | OSER1(dist=10324), EYA2(dist=157594) | 1.69E-06 |
| 23 | 3147 | 3148 | GA/G | MIR7478(dist=30701) | 9.41E-08 |
| 23 | 9467 | 9472 | CTTGTT/C | MIR7478(dist=24377) | 6.71E-06 |
| 24 | 3622894 | 3622896 | AAG/A | TECTA(dist=35100), TMEM136(dist=189854) | 5.63E-06 |
| 24 | 3622902 | 3622902 | G/GAC | TECTA(dist=35108), TMEM136(dist=189848) | 2.31E-06 |
| 25 | 2127007 | 2127026 | TGCCTTGCCTTGCCTGCCTG/T | UBQLN4(dist=576522), LOC100859191(dist=23095) | 2.04E-07 |
| 25 | 2136983 | 2136983 | G/GC | UBQLN4(dist=586498), LOC100859191(dist=13138) | 6.39E-07 |
| 26 | 5307928 | 5307968 | TAGGCAAGGCAAGGCAATGCCAGGCAAGGCAAGGCAATGCA/T | MIR7454(dist=37840) | 9.17E-07 |
| 26 | 5383079 | 5383084 | TAGGCA/T | MIR7454(dist=112991) | 4.94E-08 |
| 27 | 470370 | 470371 | TG/T | DAD1(dist=1805640) | 1.06E-08 |
| 27 | 485131 | 485131 | T/TTC | DAD1(dist=1790880) | 2.86E-08 |
| 27 | 485134 | 485176 | GCAAAGAGAAAACGGGTGATTTTCGGGTTAAAAGGGGGATTGT/GAAGAGAAAACGGGTGATTTTCGGGTTAAAAGGGGGATTGT | DAD1(dist=1790835) | 8.54E-09 |
| 27 | 487183 | 487183 | G/GAA | DAD1(dist=1788828) | 4.72E-09 |
| 27 | 517513 | 517513 | C/CG | DAD1(dist=1758498) | 1.43E-07 |
| 27 | 517757 | 517757 | C/CG | DAD1(dist=1758254) | 1.59E-07 |
| 27 | 1571476 | 1571517 | TTAAATGGGGGATTTCCGAAGTGAAACTTCATGATTTTCGCC/T | DAD1(dist=704494) | 9.73E-06 |
| 27 | 1708687 | 1708727 | AGCCTTGCCCCGTCCCACCCCACCTCGCAGCGCCCTGCCTC/A | DAD1(dist=567284) | 2.23E-11 |
| 27 | 1710692 | 1710693 | TG/T | DAD1(dist=565318) | 5.41E-06 |
| 31 | 50715 | 50716 | TA/T | - | 1.03E-06 |
| 31 | 190422 | 190422 | T/TC | - | 5.83E-09 |
| 31 | 190546 | 190581 | TAGGGTTAGGGTTAGGCCTAGGGTAAGTTTAGGGTA/T | - | 1.69E-06 |
| 31 | 199871 | 199873 | AGG/A | - | 1.37E-08 |
| 31 | 206521 | 206545 | AGGGTTAGGGTTAGGGTTAGCTTTT/A | - | 4.69E-09 |
| 31 | 214973 | 215015 | ACGCGTTTTCTCTACGAGAACCCCCATTTAAGCAAAAATCACG/ACGTTTTCTCTACGAGAACCCCCATTTAAGCAAAAATCACG | - | 2.45E-07 |
| 31 | 4646553 | 4646553 | C/CTTGTT | CHIR-A2(dist=197595), PIT54(dist=276099) | 6.01E-07 |
| 31 | 4652955 | 4652995 | CCTTGCCTTGCCTTGCCTTGCCTTGCCTTGCCTTGCCTGGA/C | CHIR-A2(dist=203997), PIT54(dist=269657) | 1.49E-11 |
| 31 | 4666297 | 4666300 | GGAC/G | CHIR-A2(dist=217339), PIT54(dist=256352) | 2.02E-06 |
| 31 | 4666301 | 4666303 | TTG/T | CHIR-A2(dist=217343), PIT54(dist=256349) | 1.67E-06 |
| 31 | 4666332 | 4666332 | A/AT | CHIR-A2(dist=217374), PIT54(dist=256320) | 3.82E-06 |
| 33 | 2994961 | 2994986 | TTTGCCTTGCCTGGCCATGCCTTGCC/T | GR42L6(dist=640713) | 1.45E-06 |
| 33 | 3545226 | 3545226 | T/TC | GR42L6(dist=90473) | 2.26E-06 |

Supplementary table 5 KEGG pathway enrichment

| Pathway | out (27) | All (6621) | p value | Candidate genes |
| --- | --- | --- | --- | --- |
| Non-alcoholic fatty liver disease (NAFLD) | 2 (7.41%) | 9 (0.14%) | 0.0006 | CASP14; MLXIPL |
| Cardiac muscle contraction | 3 (11.11%) | 70 (1.06%) | 0.0028 | CACNA1S; ATP2A3; MYH7 |
| Glycine, serine and threonine metabolism | 2 (7.41%) | 39 (0.59%) | 0.0108 | MAOA; SDSL |
| Valine, leucine and isoleucine biosynthesis | 1 (3.7%) | 3 (0.05%) | 0.0122 | SDSL |
| Apoptosis - multiple species | 1 (3.7%) | 3 (0.05%) | 0.0122 | CASP14 |
| Adrenergic signaling in cardiomyocytes | 3 (11.11%) | 132 (1.99%) | 0.016 | CACNA1S; ATP2A3; MYH7 |
| Protein processing in endoplasmic reticulum | 3 (11.11%) | 150 (2.27%) | 0.0224 | UBQLN4; CRYAB; DAD1 |
| MAPK signaling pathway | 4 (14.81%) | 271 (4.09%) | 0.0229 | GRB2; CACNA1S; RPS6KA; DUSP7 |
| TNF signaling pathway | 1 (3.7%) | 8 (0.12%) | 0.0322 | CASP14 |
| Apoptosis - fly | 1 (3.7%) | 9 (0.14%) | 0.0361 | CASP14 |
| GnRH signaling pathway | 2 (7.41%) | 84 (1.27%) | 0.0455 | GRB2; CACNA1S |
| TGF-beta signaling pathway | 2 (7.41%) | 93 (1.4%) | 0.0546 | ID2; RGMA |
| Pertussis | 1 (3.7%) | 14 (0.21%) | 0.0557 | CASP14 |
| Legionellosis | 1 (3.7%) | 14 (0.21%) | 0.0557 | CASP14 |
| Phenylalanine metabolism | 1 (3.7%) | 16 (0.24%) | 0.0634 | MAOA |
| Glycosaminoglycan biosynthesis - keratan sulfate | 1 (3.7%) | 16 (0.24%) | 0.0634 | CHST6 |
| Insulin resistance | 1 (3.7%) | 17 (0.26%) | 0.0672 | MLXIPL |
| Pathogenic Escherichia coli infection | 2 (7.41%) | 115 (1.74%) | 0.0792 | CASP14; ABCF2 |
| Histidine metabolism | 1 (3.7%) | 23 (0.35%) | 0.0898 | MAOA |
| Autophagy - animal | 2 (7.41%) | 127 (1.92%) | 0.0939 | ATG4B; SNAP29 |
| Alzheimer disease | 1 (3.7%) | 26 (0.39%) | 0.101 | CASP14 |
| Autophagy - other eukaryotes | 1 (3.7%) | 28 (0.42%) | 0.1083 | ATG4B |
| SNARE interactions in vesicular transport | 1 (3.7%) | 29 (0.44%) | 0.112 | SNAP29 |
| mTOR signaling pathway | 2 (7.41%) | 144 (2.17%) | 0.1159 | GRB2; RPS6KA |
| Tyrosine metabolism | 1 (3.7%) | 35 (0.53%) | 0.1336 | MAOA |
| Drug metabolism - cytochrome P450 | 1 (3.7%) | 36 (0.54%) | 0.1371 | MAOA |
| Signaling pathways regulating pluripotency of stem cells | 1 (3.7%) | 36 (0.54%) | 0.1371 | MYF5 |
| Basal transcription factors | 1 (3.7%) | 37 (0.56%) | 0.1407 | GTF2H5 |
| Nucleotide excision repair | 1 (3.7%) | 38 (0.57%) | 0.1442 | GTF2H5 |
| Tryptophan metabolism | 1 (3.7%) | 40 (0.6%) | 0.1512 | MAOA |
| Retinol metabolism | 1 (3.7%) | 41 (0.62%) | 0.1547 | DHRS4 |
| Cysteine and methionine metabolism | 1 (3.7%) | 45 (0.68%) | 0.1685 | SDSL |
| Arginine and proline metabolism | 1 (3.7%) | 45 (0.68%) | 0.1685 | MAOA |
| Various types of N-glycan biosynthesis | 1 (3.7%) | 46 (0.69%) | 0.1719 | DAD1 |
| Sphingolipid metabolism | 1 (3.7%) | 47 (0.71%) | 0.1753 | SGMS1 |
| Axon guidance | 1 (3.7%) | 52 (0.79%) | 0.1921 | EPHA3 |
| N-Glycan biosynthesis | 1 (3.7%) | 53 (0.8%) | 0.1954 | DAD1 |
| Salmonella infection | 2 (7.41%) | 208 (3.14%) | 0.2076 | CASP14; S100A10 |
| Biosynthesis of amino acids | 1 (3.7%) | 63 (0.95%) | 0.2279 | SDSL |
| Pathways in cancer | 1 (3.7%) | 70 (1.06%) | 0.2499 | CASP14 |
| Progesterone-mediated oocyte maturation | 1 (3.7%) | 78 (1.18%) | 0.2743 | RPS6KA |
| Peroxisome | 1 (3.7%) | 81 (1.22%) | 0.2832 | DHRS4 |
| ErbB signaling pathway | 1 (3.7%) | 82 (1.24%) | 0.2862 | GRB2 |
| Gap junction | 1 (3.7%) | 90 (1.36%) | 0.3094 | GRB2 |
| Calcium signaling pathway | 2 (7.41%) | 275 (4.15%) | 0.3098 | CACNA1S; ATP2A3 |
| Carbon metabolism | 1 (3.7%) | 99 (1.5%) | 0.3347 | SDSL |
| Oocyte meiosis | 1 (3.7%) | 100 (1.51%) | 0.3375 | RPS6KA |
| Vascular smooth muscle contraction | 1 (3.7%) | 122 (1.84%) | 0.3954 | CACNA1S |
| FoxO signaling pathway | 1 (3.7%) | 125 (1.89%) | 0.4029 | GRB2 |
| Apoptosis | 1 (3.7%) | 129 (1.95%) | 0.4127 | CASP14 |
| Insulin signaling pathway | 1 (3.7%) | 129 (1.95%) | 0.4127 | GRB2 |
| Influenza A | 1 (3.7%) | 154 (2.33%) | 0.471 | RSAD2 |
| Focal adhesion | 1 (3.7%) | 199 (3.01%) | 0.562 | GRB2 |
| Metabolic pathways | 5 (18.52%) | 1379 (20.83%) | 0.6906 | MAOA; SGMS1; DHRS4; DAD1; SDSL |
| Neuroactive ligand-receptor interaction | 1 (3.7%) | 344 (5.2%) | 0.7639 | NTS |


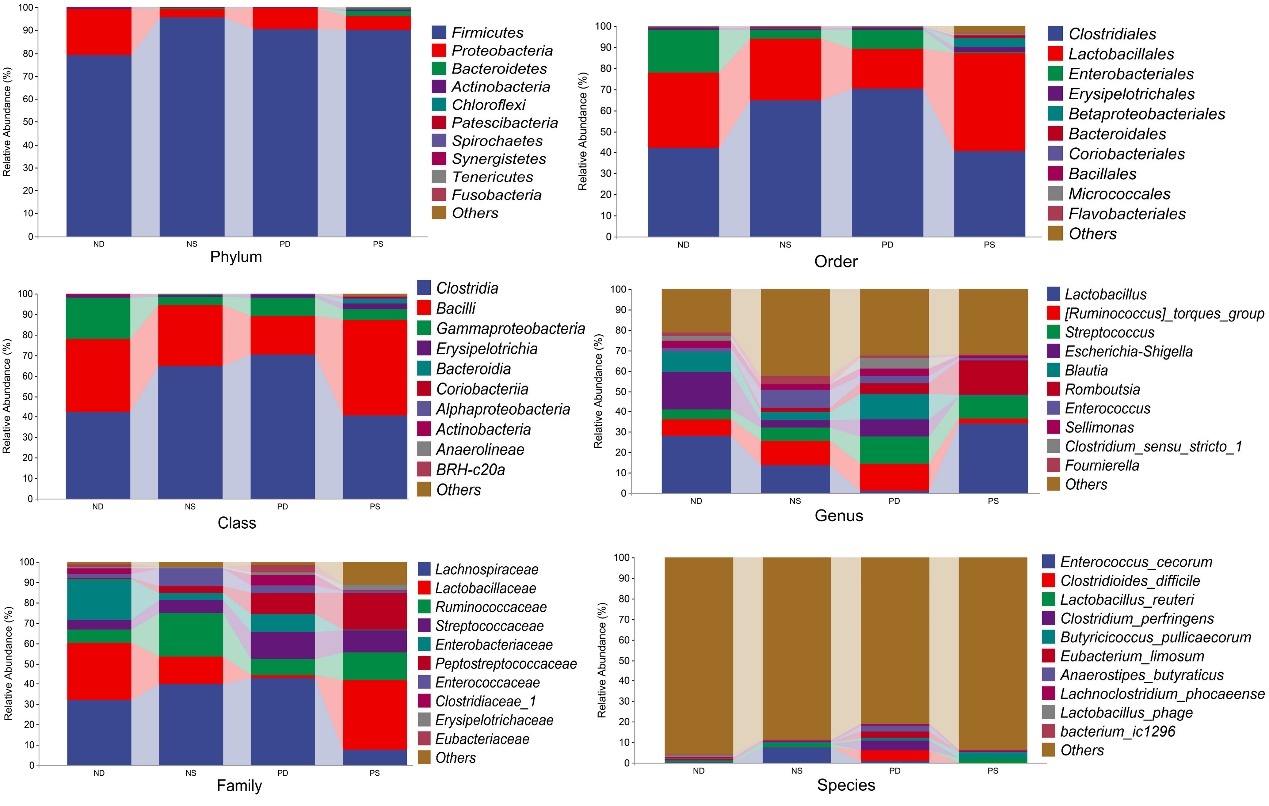


Supplementary figure 5 The composition and distribution of cecal microorganisms in each group infected with *Salmonella pullorum* at different classification levels

Note: Only the top 10 bacteria are shown.

Supplementary table 6 Analysis of significant different bacteria between groups

| Significant different bacteria | abundance | group | LDA score | p value |
| --- | --- | --- | --- | --- |
| Actinobacteria. Coriobacteriia. Coriobacteriales. Eggerthellaceae. Slackia | 2.64 | PS | 3.03 | 1.77E-05 |
| Actinobacteria. Coriobacteriia. Coriobacteriales. Eggerthellaceae. Slackia. Enterorhabdus_sp_ | 2.64 | PS | 3.03 | 1.77E-05 |
| Proteobacteria. Gammaproteobacteria. Enterobacteriales. Enterobacteriaceae. Escherichia_Shigella | 5.27 | ND | 5.00 | 2.98E-05 |
| Proteobacteria. Alphaproteobacteria. Rhizobiales. Rhizobiaceae. Ochrobactrum | 2.27 | PD | 3.84 | 3.34E-05 |
| Proteobacteria. Gammaproteobacteria. Pseudomonadales. Moraxellaceae. Acinetobacter. Acinetobacter_harbinensis | 2.36 | PS | 3.32 | 4.32E-05 |
| Proteobacteria. Gammaproteobacteria. Enterobacteriales. Enterobacteriaceae. Enterobacter | 2.39 | ND | 2.70 | 4.70E-05 |
| Firmicutes. Clostridia. Clostridiales. Lachnospiraceae. CHKCI001 | 3.32 | NS | 3.14 | 5.45E-05 |
| Proteobacteria. Gammaproteobacteria. Betaproteobacteriales. Hydrogenophilaceae. Thiobacillus | 2.35 | PD | 2.92 | 0.0001 |
| Proteobacteria. Gammaproteobacteria. Enterobacteriales. Enterobacteriaceae. Escherichia_Shigella. Klebsiella_pneumoniae | 3.31 | ND | 3.08 | 0.0001 |
| Firmicutes | 5.98 | NS | 4.95 | 0.0002 |
| Proteobacteria | 5.31 | ND | 4.95 | 0.0002 |
| Proteobacteria. Gammaproteobacteria. Enterobacteriales. Enterobacteriaceae. Klebsiella | 3.73 | ND | 3.48 | 0.0002 |
| Proteobacteria. Gammaproteobacteria. Betaproteobacteriales. Hydrogenophilaceae | 2.35 | PD | 3.02 | 0.0003 |
| Firmicutes. Clostridia. Clostridiales. Ruminococcaceae. Ruminococcaceae_UCG_013 | 3.98 | NS | 3.56 | 0.0006 |
| Firmicutes. Clostridia. Clostridiales. Ruminococcaceae. Ruminococcus_2 | 3.12 | PS | 3.18 | 0.0008 |
| Firmicutes. Clostridia. Clostridiales. Eubacteriaceae. Eubacterium. Eubacterium_limosum | 4.52 | PD | 4.21 | 0.0009 |
| Actinobacteria. Actinobacteria. Micrococcales. Beutenbergiaceae | 2.44 | NS | 2.83 | 0.0010 |
| Actinobacteria. Actinobacteria. Micrococcales. Beutenbergiaceae. Salana | 2.44 | NS | 2.81 | 0.0010 |
| Firmicutes. Clostridia. Clostridiales. Lachnospiraceae. _Eubacterium__hallii_group | 3.44 | NS | 3.17 | 0.0011 |
| Firmicutes. Clostridia. Clostridiales. Lachnospiraceae. Tyzzerella_3 | 3.31 | NS | 2.98 | 0.0012 |
| Firmicutes. Clostridia. Clostridiales. Eubacteriaceae. Eubacterium | 4.54 | PD | 4.23 | 0.0012 |
| Firmicutes. Bacilli. Lactobacillales. Lactobacillaceae. Lactobacillus. Lactobacillus_vaginalis | 3.33 | PS | 3.06 | 0.0013 |
| Proteobacteria. Gammaproteobacteria. Enterobacteriales. Enterobacteriaceae. Escherichia_Shigella. Streptomyces_sp_ | 2.80 | ND | 2.65 | 0.0016 |
| Firmicutes. Clostridia. Clostridiales. Eubacteriaceae | 4.55 | PD | 4.24 | 0.0021 |
| Bacteroidetes. Bacteroidia. Bacteroidales. Dysgonomonadaceae. Dysgonomonas | 2.15 | NS | 3.11 | 0.0025 |
| Actinobacteria. Actinobacteria. Corynebacteriales. Corynebacteriaceae. Corynebacterium_1 | 2.07 | PS | 3.49 | 0.0028 |
| Actinobacteria. Coriobacteriia. Coriobacteriales. Eggerthellaceae. Gordonibacter. Gordonibacter_urolithinfaciens | 3.06 | ND | 2.86 | 0.0031 |
| Firmicutes. Clostridia. Clostridiales. Ruminococcaceae. Butyricicoccus. Butyricicoccus_pullicaecorum | 4.40 | PS | 4.09 | 0.0038 |
| Proteobacteria. Gammaproteobacteria. Oceanospirillales | 1.73 | PS | 3.47 | 0.0039 |
| Proteobacteria. Gammaproteobacteria. Pseudomonadales. Pseudomonadaceae | 3.04 | PS | 2.90 | 0.0041 |
| Proteobacteria. Gammaproteobacteria. Pseudomonadales. Pseudomonadaceae. Pseudomonas | 3.04 | PS | 2.90 | 0.0041 |
| Proteobacteria. Gammaproteobacteria. Pseudomonadales | 3.17 | PS | 2.92 | 0.0042 |
| Actinobacteria. Coriobacteriia. Coriobacteriales. Eggerthellaceae. CHKCI002. Coriobacteriaceae_bacterium | 3.45 | PD | 3.10 | 0.0043 |
| Actinobacteria. Coriobacteriia. Coriobacteriales. Eggerthellaceae. CHKCI002 | 3.45 | PD | 3.10 | 0.0043 |
| Firmicutes. Bacilli. Lactobacillales. Enterococcaceae. Enterococcus. Enterococcus_cecorum | 4.87 | NS | 4.46 | 0.0054 |
| Firmicutes. Clostridia. Clostridiales. Lachnospiraceae. Sellimonas. Lachnoclostridium_phocaeense | 3.78 | NS | 3.33 | 0.0056 |
| Proteobacteria. Gammaproteobacteria. Pseudomonadales. Moraxellaceae. Acinetobacter | 2.54 | PS | 2.88 | 0.0066 |
| Proteobacteria. Gammaproteobacteria. Pseudomonadales. Moraxellaceae | 2.59 | PS | 2.81 | 0.0074 |
| Actinobacteria. Actinobacteria. Corynebacteriales. Corynebacteriaceae | 2.07 | PS | 3.75 | 0.0077 |
| Actinobacteria. Actinobacteria. Corynebacteriales | 2.17 | PS | 3.79 | 0.0077 |
| Firmicutes. Bacilli. Lactobacillales. Lactobacillaceae. Lactobacillus. Lactobacillus_rennini | 2.51 | NS | 2.53 | 0.0095 |
| Firmicutes. Clostridia. Clostridiales. Ruminococcaceae. CAG_352 | 2.26 | PS | 3.49 | 0.0101 |
| Proteobacteria. Gammaproteobacteria. Aeromonadales | 3.12 | PS | 2.93 | 0.0110 |
| Firmicutes. Clostridia. Clostridiales. Lachnospiraceae. Anaerostipes. Anaerostipes_caccae | 3.49 | ND | 3.20 | 0.0120 |
| Firmicutes. Erysipelotrichia. Erysipelotrichales. Erysipelotrichaceae. Erysipelatoclostridium. bacterium_ic1391 | 3.34 | PS | 3.01 | 0.0130 |
| Firmicutes. Clostridia. Clostridiales. Lachnospiraceae. Eisenbergiella | 4.06 | NS | 3.74 | 0.0174 |
| Actinobacteria. Coriobacteriia. Coriobacteriales. Eggerthellaceae. Gordonibacter | 3.28 | ND | 2.91 | 0.0190 |
| Cyanobacteria. Oxyphotobacteria. Chloroplast. Chloroplast. Chloroplast | 2.19 | NS | 2.46 | 0.0272 |
| Cyanobacteria. Oxyphotobacteria. Chloroplast | 2.19 | NS | 2.47 | 0.0272 |
| Cyanobacteria. Oxyphotobacteria. Chloroplast. Chloroplast | 2.19 | NS | 2.44 | 0.0272 |
| Firmicutes. Bacilli. Lactobacillales. Lactobacillaceae. Lactobacillus. Tetragenococcus_halophilus | 2.89 | ND | 2.73 | 0.0428 |

Supplementary table 7 Significant different bacteria between NS and ND at the genus level

| Genus | ND | | NS | | p value |
| --- | --- | --- | --- | --- | --- |
|  | mean | standard deviation | mean | standard deviation |  |
| *g__Lactobacillus* | 12.01 | 2.14 | 10.39 | 2.55 | 0.04 |
| *g__Escherichia-Shigella* | 11.97 | 1.55 | 8.28 | 2.41 | 1.97E-06 |
| *g__[Ruminococcus]_torques_group* | 9.64 | 2.55 | 11.25 | 1.46 | 0.02 |
| *g__unclassified_Ruminococcaceae* | 6.99 | 2.15 | 9.49 | 1.43 | 0.0002 |
| *g__Fournierella* | 6.28 | 3.70 | 8.73 | 2.38 | 0.02 |
| *g__unclassified_Enterobacteriaceae* | 6.12 | 2.49 | 3.09 | 1.84 | 0.0001 |
| *g__Butyricicoccus* | 5.87 | 2.71 | 7.77 | 1.63 | 0.01 |
| *g__[Clostridium]_innocuum_group* | 5.81 | 2.29 | 3.81 | 2.00 | 0.007 |
| *g__Anaerostipes* | 5.68 | 2.90 | 3.06 | 2.75 | 0.007 |
| *g__Negativibacillus* | 5.61 | 3.02 | 7.80 | 1.44 | 0.007 |
| *g__[Eubacterium]_coprostanoligenes_group* | 5.18 | 2.97 | 7.22 | 1.16 | 0.008 |
| *g__Eisenbergiella* | 5.12 | 3.46 | 7.63 | 1.60 | 0.007 |
| *g__Klebsiella* | 4.83 | 2.86 | 1.51 | 1.99 | 0.0002 |
| *g__Gordonibacter* | 4.74 | 2.28 | 2.94 | 1.84 | 0.01 |
| *g__Caproiciproducens* | 4.46 | 2.57 | 6.52 | 1.16 | 0.003 |
| *g__Lachnospiraceae_NC2004_group* | 4.44 | 2.13 | 6.28 | 2.29 | 0.01 |
| *g__[Ruminococcus]_gauvreauii_group* | 4.32 | 3.34 | 6.84 | 1.45 | 0.004 |
| *g__Romboutsia* | 4.30 | 1.83 | 7.98 | 2.21 | 2.02E-06 |
| *g__Tyzzerella* | 3.91 | 3.50 | 5.79 | 2.04 | 0.05 |
| *g__Ruminococcaceae_UCG-013* | 3.48 | 3.16 | 7.46 | 2.05 | 4.69E-05 |
| *g__[Eubacterium]_oxidoreducens_group* | 3.32 | 2.21 | 4.91 | 1.68 | 0.02 |
| *g__uncultured* | 3.17 | 1.98 | 6.40 | 1.48 | 1.5E-06 |
| *g__Aeromonas* | 3.03 | 2.12 | 1.18 | 1.86 | 0.007 |
| *g__unclassified_Clostridiales* | 2.98 | 2.30 | 5.29 | 2.30 | 0.004 |
| *g__unclassified_Firmicutes* | 2.81 | 1.36 | 5.04 | 1.57 | 3.68E-05 |
| *g__Bacillus* | 2.79 | 2.06 | 5.76 | 1.44 | 8.6E-06 |
| *g__Enterobacter* | 2.20 | 1.60 | 0.44 | 1.10 | 0.0003 |
| *g__Pseudoflavonifractor* | 2.19 | 2.51 | 4.95 | 2.64 | 0.002 |
| *g__uncultured* | 1.99 | 1.73 | 4.17 | 2.71 | 0.005 |
| *g__Anaerofustis* | 1.79 | 2.43 | 3.94 | 1.65 | 0.003 |
| *g__Clostridiales_vadinBB60_group* | 1.63 | 1.97 | 4.67 | 1.43 | 3.41E-06 |
| *g__unclassified_Peptostreptococcaceae* | 1.61 | 2.09 | 5.62 | 2.85 | 1.58E-05 |
| *g__Ruminococcaceae_UCG-004* | 1.59 | 1.80 | 3.87 | 3.09 | 0.009 |
| *g__Subdoligranulum* | 1.59 | 2.34 | 8.22 | 3.28 | 1.44E-08 |
| *g__CHKCI002* | 1.58 | 3.22 | 3.65 | 1.72 | 0.02 |
| *g__CHKCI001* | 1.45 | 1.47 | 4.58 | 1.95 | 2.1E-06 |
| *g__Citrobacter* | 1.45 | 1.83 | 0.00 | 0.00 | 0.001 |
| *g__ASF356* | 1.25 | 1.69 | 2.69 | 2.42 | 0.04 |
| *g__GCA-900066225* | 1.23 | 2.33 | 5.26 | 2.10 | 2.02E-06 |
| *g__Tyzzerella_3* | 1.22 | 2.18 | 4.45 | 2.56 | 0.0002 |
| *g__Ruminococcaceae_UCG-014* | 1.09 | 2.19 | 3.51 | 3.67 | 0.02 |
| *g__Allorhizobium-Neorhizobium-Pararhizobium-Rhizobium* | 0.95 | 1.26 | 0.00 | 0.00 | 0.002 |
| *g__Acetobacter* | 0.93 | 1.63 | 0.00 | 0.00 | 0.02 |
| *g__Intestinimonas* | 0.91 | 1.02 | 3.61 | 2.44 | 7.07E-05 |
| *g__uncultured* | 0.89 | 1.95 | 4.82 | 3.35 | 7.97E-05 |
| *g__Dysgonomonas* | 0.88 | 1.18 | 2.02 | 1.39 | 0.009 |
| *g__GCA-900066575* | 0.76 | 1.54 | 4.52 | 2.27 | 6.24E-07 |
| *g__Defluviitaleaceae_UCG-011* | 0.66 | 1.30 | 2.13 | 1.53 | 0.003 |
| *g__uncultured* | 0.65 | 1.26 | 2.78 | 2.72 | 0.004 |
| *g__Gluconobacter* | 0.64 | 1.32 | 0.00 | 0.00 | 0.04 |
| *g__[Eubacterium]_nodatum_group* | 0.59 | 1.22 | 1.80 | 1.86 | 0.02 |
| *g__unclassified_Bacilli* | 0.59 | 0.92 | 1.85 | 1.07 | 0.0004 |
| *g__Shuttleworthia* | 0.59 | 1.28 | 3.17 | 3.42 | 0.004 |
| *g__Anaerofilum* | 0.51 | 1.40 | 3.83 | 2.57 | 1.62E-05 |
| *g__unclassified_Clostridiaceae_1* | 0.45 | 0.78 | 0.00 | 0.00 | 0.02 |
| *g__Lachnospiraceae_FE2018_group* | 0.41 | 1.47 | 1.97 | 2.82 | 0.04 |
| *g__[Eubacterium]_hallii_group* | 0.37 | 1.39 | 3.71 | 3.44 | 0.0003 |
| *g__Mollicutes_RF39* | 0.28 | 1.22 | 1.70 | 2.63 | 0.04 |
| *g__Erysipelotrichaceae* | 0.21 | 0.65 | 1.34 | 2.38 | 0.05 |
| *g__Pygmaiobacter* | 0.17 | 0.54 | 1.47 | 1.90 | 0.007 |
| *g__Lachnospiraceae_UCG-010* | 0.16 | 0.69 | 1.05 | 1.47 | 0.02 |
| *g__unclassified_Bacillales* | 0.13 | 0.40 | 0.71 | 1.12 | 0.04 |
| *g__unclassified_Bacillaceae* | 0.08 | 0.35 | 0.84 | 1.41 | 0.03 |
| *g__Shewanella* | 0.00 | 0.00 | 1.45 | 2.15 | 0.006 |
| *g__Thermovirga* | 0.00 | 0.00 | 0.80 | 1.20 | 0.006 |
| *g__UC5-1-2E3* | 0.00 | 0.00 | 1.68 | 2.59 | 0.007 |
| *g__Halomonas* | 0.00 | 0.00 | 0.23 | 0.47 | 0.04 |
| *g__Slackia* | 0.00 | 0.00 | 0.68 | 1.48 | 0.05 |

Supplementary table 8 Significant different bacteria between ND and PD at the genus level

| Genus | ND(n=20) | | PD(n=5) | | p value |
| --- | --- | --- | --- | --- | --- |
|  | mean | standard deviation | mean | standard deviation |  |
| *Lactobacillus* | 12.01 | 2.14 | 7.65 | 1.71 | 0.0005 |
| *unclassified_Enterobacteriaceae* | 6.12 | 2.49 | 3.11 | 3.00 | 0.04 |
| *Lachnospiraceae_NK4A136_group* | 4.12 | 2.71 | 1.40 | 1.88 | 0.05 |
| *Aeromonas* | 3.03 | 2.12 | 0.00 | 0.00 | 0.0055 |
| *Enterobacter* | 2.20 | 1.60 | 0.00 | 0.00 | 0.0073 |
| *Eubacterium* | 2.07 | 3.94 | 8.49 | 2.13 | 0.0026 |
| *Muribaculaceae* | 1.93 | 1.41 | 0.20 | 0.40 | 0.02 |
| *unclassified_Gammaproteobacteria* | 1.86 | 0.78 | 0.52 | 0.66 | 0.0024 |
| *Salana* | 1.65 | 1.33 | 0.00 | 0.00 | 0.01 |
| *CHKCI002* | 1.58 | 3.22 | 5.77 | 1.65 | 0.01 |
| *Desulfovibrio* | 1.11 | 1.16 | 0.00 | 0.00 | 0.05 |
| *uncultured* | 0.65 | 1.26 | 4.20 | 2.90 | 0.0006 |
| *Thiobacillus* | 0.10 | 0.44 | 2.83 | 0.53 | 5E-11 |
| *Ochrobactrum* | 0.08 | 0.35 | 2.62 | 1.32 | 1.92E-07 |
| *Slackia* | 0.00 | 0.00 | 0.32 | 0.63 | 0.04 |
| *unclassified_Lactobacillaceae* | 0.00 | 0.00 | 0.20 | 0.40 | 0.04 |

Supplementary table 9 Significant different bacteria between PS and PD at the genus level

| Genus | PD(n=5) | | PS(n=5) | | p value |
| --- | --- | --- | --- | --- | --- |
|  | mean | standard deviation | mean | standard deviation |  |
| *Escherichia-Shigella* | 10.63 | 1.73 | 4.73 | 3.09 | 0.01 |
| *Eubacterium* | 8.49 | 2.13 | 3.96 | 2.96 | 0.04 |
| *Caproiciproducens* | 3.24 | 2.07 | 7.28 | 1.58 | 0.01 |
| *unclassified_Firmicutes* | 3.03 | 1.10 | 5.12 | 0.98 | 0.02 |
| *Cetobacterium* | 2.89 | 1.49 | 0.46 | 0.93 | 0.02 |
| *Thiobacillus* | 2.83 | 0.53 | 0.40 | 0.80 | 0.0009 |
| *Ochrobactrum* | 2.62 | 1.32 | 0.60 | 0.80 | 0.03 |
| *Acinetobacter* | 1.63 | 0.88 | 3.40 | 0.71 | 0.01 |
| *Clostridium_sensu_stricto_12* | 1.18 | 0.99 | 0.00 | 0.00 | 0.04 |
| *Pseudomonas* | 0.86 | 1.06 | 4.58 | 1.19 | 0.0016 |
| *Slackia* | 0.32 | 0.63 | 3.24 | 1.53 | 0.0076 |
| *Weissella* | 0.00 | 0.00 | 2.37 | 2.01 | 0.05 |
| *unclassified_Actinobacteria* | 0.00 | 0.00 | 0.92 | 0.81 | 0.05 |

Supplementary table 10 Significant different bacteria between NS and PS at the genus level

| Genus | NS(n=20) | | PS(n=5) | | p value |
| --- | --- | --- | --- | --- | --- |
|  | mean | standard deviation | mean | standard deviation |  |
| *unclassified_Lachnospiraceae* | 11.33 | 1.68 | 7.81 | 1.94 | 0.0007 |
| *[Ruminococcus]_torques_group* | 11.25 | 1.46 | 8.84 | 1.67 | 0.005 |
| *Lachnoclostridium* | 8.73 | 1.40 | 5.71 | 1.57 | 0.0005 |
| *Eisenbergiella* | 7.63 | 1.60 | 4.62 | 1.15 | 0.0009 |
| *Bacillus* | 5.76 | 1.44 | 2.89 | 2.37 | 0.003 |
| *[Eubacterium]_oxidoreducens_group* | 4.91 | 1.68 | 1.41 | 1.39 | 0.0004 |
| *CHKCI001* | 4.58 | 1.95 | 1.32 | 1.27 | 0.002 |
| *Salana* | 2.59 | 1.68 | 0.00 | 0.00 | 0.003 |
| *Pseudomonas* | 2.35 | 1.40 | 4.58 | 1.19 | 0.004 |
| *Acinetobacter* | 1.11 | 1.23 | 3.40 | 0.71 | 0.0009 |
| *Slackia* | 0.68 | 1.48 | 3.24 | 1.53 | 0.003 |
| *unclassified_Lactobacillaceae* | 0.19 | 0.64 | 1.63 | 1.48 | 0.004 |
| *Corynebacterium_1* | 0.18 | 0.54 | 1.60 | 1.45 | 0.003 |
| *Ruminococcus_2* | 0.10 | 0.44 | 2.93 | 2.81 | 0.0004 |
| *Acetobacter* | 0.00 | 0.00 | 1.25 | 1.61 | 0.003 |
| *Ochrobactrum* | 0.00 | 0.00 | 0.60 | 0.80 | 0.004 |
| *unclassified_Clostridiaceae_1* | 0.00 | 0.00 | 0.66 | 0.91 | 0.005 |

Supplementary table 11 Differential bacteria between P and PS

| Genus | P-mean | P- standard deviation | PS-mean | PS- standard deviation | p value |
| --- | --- | --- | --- | --- | --- |
| *Escherichia-Shigella* | 3508 | 3946 | 119 | 143 | 4.15E-06 |
| *Bacteroides* | 726 | 2003 | 0 | 0 | 0.029 |
| *Candidatus_Arthromitus* | 626 | 853 | 0 | 0 | 4.63E-05 |
| *Helicobacter* | 429 | 914 | 0 | 0 | 0.006 |
| *Faecalibacterium* | 364 | 920 | 2 | 3 | 0.018 |
| *uncultured* | 219 | 527 | 0 | 0 | 0.013 |
| *Megamonas* | 170 | 410 | 0 | 0 | 0.014 |
| *Gallibacterium* | 94 | 214 | 0 | 0 | 0.009 |
| *uncultured* | 68 | 148 | 0 | 0 | 0.006 |
| *Erysipelatoclostridium* | 21 | 78 | 206 | 91 | 0.013 |
| *uncultured* | 21 | 62 | 0 | 0 | 0.043 |
| *Phascolarctobacterium* | 15 | 44 | 0 | 0 | 0.037 |
| *Lachnospiraceae_FCS020_group* | 11 | 25 | 0 | 0 | 0.013 |
| *Haemophilus* | 9 | 26 | 0 | 0 | 0.037 |
| *unidentified* | 9 | 22 | 0 | 0 | 0.018 |
| *Coprococcus_1* | 8 | 17 | 0 | 0 | 0.006 |
| *Isobaculum* | 7 | 18 | 0 | 0 | 0.022 |
| *Veillonella* | 7 | 11 | 0 | 0 | 0.0005 |
| *Ruminococcaceae_UCG-005* | 6 | 17 | 0 | 0 | 0.030 |
| *Butyricimonas* | 6 | 15 | 0 | 0 | 0.029 |
| *Massilia* | 6 | 16 | 0 | 0 | 0.035 |
| *Ochrobactrum* | 5 | 9 | 1 | 1 | 0.016 |
| *Sutterella* | 4 | 11 | 0 | 0 | 0.023 |
| *Lachnospiraceae_FE2018_group* | 4 | 8 | 0 | 0 | 0.006 |
| *unidentified* | 4 | 11 | 0 | 0 | 0.035 |
| *Bilophila* | 3 | 8 | 0 | 0 | 0.011 |
| *Acinetobacter* | 3 | 6 | 11 | 4 | 0.018 |
| *Porphyromonas* | 3 | 7 | 0 | 0 | 0.010 |
| *Rothia* | 3 | 4 | 0 | 0 | 0.0002 |
| *uncultured_bacterium* | 3 | 8 | 0 | 0 | 0.047 |
| *Ruminococcus_1* | 2 | 5 | 0 | 0 | 0.027 |
| *Catellicoccus* | 2 | 7 | 0 | 0 | 0.040 |
| *Synergistes* | 2 | 5 | 0 | 0 | 0.019 |
| *Mucilaginibacter* | 2 | 5 | 0 | 0 | 0.044 |
| *Ruminococcaceae_UCG-013* | 1 | 5 | 103 | 68 | 0.041 |
| *uncultured* | 1 | 4 | 0 | 0 | 0.045 |
| *Ruminococcaceae_UCG-010* | 1 | 3 | 0 | 0 | 0.025 |
| *Enorma* | 1 | 4 | 0 | 0 | 0.047 |
| *Anaerofustis* | 1 | 2 | 27 | 12 | 0.011 |
| *Family_XIII_UCG-001* | 1 | 2 | 0 | 0 | 0.038 |
| *Anaerosporobacter* | 1 | 2 | 0 | 0 | 0.039 |
| *Enterobacter* | 1 | 2 | 0 | 0 | 0.013 |
| *Asticcacaulis* | 1 | 2 | 0 | 0 | 0.021 |
| *uncultured_bacterium* | 1 | 1 | 0 | 0 | 0.021 |
| *Victivallis* | 1 | 2 | 0 | 0 | 0.032 |
| *Corynebacterium* | 0 | 1 | 0 | 0 | 0.017 |
| *Caulobacter* | 0 | 1 | 0 | 0 | 0.031 |
| *Serratia* | 0 | 1 | 0 | 0 | 0.044 |
| *uncultured* | 0 | 0 | 139 | 67 | 0.014 |
| *unclassified_Ruminococcaceae* | 0 | 0 | 588 | 289 | 0.015 |
| *Fournierella* | 0 | 0 | 127 | 69 | 0.021 |
| *unclassified_Dysgonomonadaceae* | 0 | 0 | 3 | 2 | 0.021 |
| *unclassified_Firmicutes* | 0 | 0 | 42 | 24 | 0.026 |
| *unclassified_Lactobacillales* | 0 | 0 | 105 | 75 | 0.048 |
